# SLAMF8 Downregulates Mouse Macrophage Microbicidal Mechanisms *via* PI3K Pathways

**DOI:** 10.3389/fimmu.2022.910112

**Published:** 2022-06-28

**Authors:** Salvador Romero-Pinedo, Domingo I. Rojas Barros, María José Ruiz-Magaña, Elena Maganto-García, Laura Moreno de Lara, Francisco Abadía-Molina, Cox Terhorst, Ana C. Abadía-Molina

**Affiliations:** ^1^Unidad de Inmunología, Instituto de Biopatología y Medicina Regenerativa (IBIMER), Centro de Investigación Biomédica (CIBM), Universidad de Granada, Granada, Spain; ^2^Centro de Biología Molecular “Severo Ochoa” Consejo Superior de Investigaciones Científicas-Universidad Autónoma de Madrid (CSIC-UAM), Universidad Autónoma de Madrid, Cantoblanco, Madrid, Spain; ^3^Departamento de Biología Celular, Facultad de Ciencias, Universidad de Granada, Granada, Spain; ^4^Instituto de Nutrición Y Tecnología de los Alimentos “José Mataix”, (INYTIA), Centro de Investigación Biomédica (CIBM), Universidad de Granada, Granada, Spain; ^5^Division of Immunology, Beth Israel Deaconess Medical Center, Harvard Medical School, Boston, MA, United States; ^6^Departamento de Bioqu´ımica y Biolog´ıa Molecular III e Inmunolog´ıa, Facultad de Medicina, Universidad de Granada, Granada, Spain

**Keywords:** SLAMF8, macrophages, SLAMF, PI3K signaling pathway, *Salmonella typhimurium*

## Abstract

Signaling lymphocytic activation molecule family 8 (SLAMF8) is involved in the negative modulation of NADPH oxidase activation. However, the impact of SLAMF8 downregulation on macrophage functionality and the microbicide mechanism remains elusive. To study this in depth, we first analyzed NADPH oxidase activation pathways in wild-type and SLAMF8-deficient macrophages upon different stimulus. Herein, we describe increased phosphorylation of the Erk1/2 and p38 MAP kinases, as well as increased phosphorylation of NADPH oxidase subunits in SLAMF8-deficient macrophages. Furthermore, using specific inhibitors, we observed that specific PI3K inhibition decreased the differences observed between wild-type and SLAMF8-deficient macrophages, stimulated with either PMA, LPS, or *Salmonella typhimurium* infection. Consequently, SLAMF8-deficient macrophages also showed increased recruitment of small GTPases such as Rab5 and Rab7, and the p47^phox^ subunit to cytoplasmic *Salmonella*, suggesting an impairment of *Salmonella-*containing vacuole (SCV) progression in SLAMF8-deficient macrophages. Enhanced iNOS activation, NO production, and IL-6 expression were also observed in the absence of SLAMF8 upon *Salmonella* infection, either *in vivo* or *in vitro*, while overexpression of SLAMF8 in RAW264.7 macrophages showed the opposite phenotype. In addition, SLAMF8-deficient macrophages showed increased activation of Src kinases and reduced SHP-1 phosphate levels upon IFNγ and *Salmonella* stimuli in comparison to wild-type macrophages. In agreement with *in vitro* results, *Salmonella* clearance was augmented in SLAMF8-deficient mice compared to that in wild-type mice. Therefore, in conclusion, SLAMF8 intervention upon bacterial infection downregulates mouse macrophage activation, and confirmed that SLAMF8 receptor could be a potential therapeutic target for the treatment of severe or unresolved inflammatory conditions.

## Introduction

Receptors of the signaling lymphocyte activation molecule family (SLAMF) are adhesion molecules that are differentially expressed on hematopoietic cell membranes. These receptors are involved in the modulation of specific and innate cell functions (1, 2). The SLAM family member 8 (SLAMF8/CD353/BLAME), similar to other family members, is a type I cell surface glycoprotein containing two immunoglobulin domains (IgV and IgC2), and clusters on chromosome 1q21. Its expression is induced by different stimuli, such as bacteria and IFNγ in neutrophils, macrophages (Mø), and dendritic cells. Like other SLAMF receptors, SLAMF8 has been shown to be a self-ligand receptor (3). Previously, we described that SLAMF8-deficient mice (SLAMF8^-/-^) showed enhanced protein kinase C (PKC)-mediated phosphorylation of p40^phox^ and increased reactive oxygen species (ROS) production (3, 4). In consequence, myeloid cell adhesion and migration activity were augmented in SLAMF8-deficient mice. All these data are indicative of increased activation of macrophages in the absence of SLAMF8. However, the impact of SLAMF8 deletion in microbicidal activities has not yet been described.

The microbicidal activity of ROS produced by phagocyte NADPH oxidase (NOX2) is crucial for an effective immune response and needs to be tightly regulated to ensure innate cell functionality (5). This multiprotein complex enzyme consists of cytosolic proteins p47^phox^, p67^phox^, p40^phox^, the small G-protein subfamily Rac, and the associated membrane proteins gp91^phox^ and p22^phox^ (forming the flavocytochrome b_558_) (5, 6). In resting cells, the cytosolic components p47^phox^, p67^phox^, and p40^phox^ are associated with each other, while Rac GTPase is strategically separated to avoid ROS production (7, 8). Once cells encounter a pathogen, the NOX2 subunits are activated and then recruited to membrane-bound components to produce superoxide radical anions $\left({\text{O}}_{2}{}_{-}^{•}\right)$ (9, 10). The complete activation of NOX2 is dependent on various transduction pathways and kinases (5). Specifically, NOX2 subunits are phosphorylated on different motifs directly by PKC (11–17), p38 mitogen-activated protein kinase (MAPK), and ERK1/2 (14, 18, 19). On the other hand, the dependence of NOX2 activation on the phosphatidylinositol-4,5-bisphosphate 3-kinase (PI3K) pathway has been demonstrated (20), particularly through the phosphorylation of p47^phox^ by AKT and IRAK-4 (21–23). Similarly, the Rho GTPase Rac is crucial for NOX2 activation and assembly of the cytosolic components at the phagosome membrane (24), and has been shown to be dependent on p38 MAPK activation *via* the PI3K pathway (25).

Given that SLAMF8-deficient (SLAMF8^-/-^) Mø showed early and significant superoxide $\left({\text{O}}_{2}{}_{-}^{•}\right)$ production compared to wild-type (*wt or SLAMF8^+/+^*) Mø, we postulated that SLAMF8 might mediate a general primary signal modulation not only decreasing NOX2 activation through different pathways, but also downmodulating Mø activation and bacterial clearance. To address this question, we studied NOX2 activation pathways with different stimuli and upon *Salmonella typhimurium* infection. *S. typhimurium* is a Gram-negative facultative intracellular pathogen that is the major cause of human gastroenteritis. This pathogen is used in a mouse model of human typhoid fever (26). *S. typhimurium* is able to modify the host cell microbicidal activity by inhibiting NOX2 and inducible nitric oxide synthase (iNOS) activation, and reducing production of their derivatives (27, 28). Moreover, *S. typhimurium* also prevents phagolysosome fusion and regulates the pro-inflammatory response through translocation of effector proteins into host cells or modulating the environment by type III secretion apparatus (T3SS) (29, 30). In order to determine the implication of SLAMF8 in microbicidal mechanism, in this study, we analyze the kinase-dependent activation of NOX2, and other microbicidal mechanism in SLAMF8^-/-^ macrophages and mice. This study demonstrates the SLAMF8 modulation of macrophage activation either by agonist stimulus or during *Salmonella* infection.

## Materials and Methods

### Mice

The protocol for the generation of *Slamf8*-deficient (SLAMF8^-/-^) BALB/c mice has been described previously (4). Age- and sex-matched wild-type (*wt*) BALB/c mice were purchased from Harlan Laboratories Inc. (Indianapolis, IN, USA). Mice were maintained in the animal facility (Centro de Investigación Biomédica, CIBM, and Universidad de Granada) under specific pathogen-free (SPF) conditions before use. All experimental procedures were conducted according to the *RD 53/2013* (BOE, 34, 11370-11421, 2013) and the protocols were approved by the Ethics Committee of Animal Experimentation, University of Granada (References: CEEA-379 y CEEA-417-2012). All *in vivo* experiments were performed with good animal practices according to the guidelines of the relevant local and national animal welfare bodies.

### Cells and Bacteria

#### Isolation of Thioglycollate-Elicited Peritoneal Macrophages and Culture Conditions

Macrophages were obtained as described previously (4). TGC peritoneal macrophages (pMø) were obtained from mice after intraperitoneal (i.p.) injection with 4% Difco™ Fluid TGC Medium [Becton, Dickinson and Company (BD), Franklin Lakes, NJ, USA], and maintained in HyClone RPMI 1640 medium (GE Healthcare, Chicago, IL, USA), supplemented with 0.5% fetal bovine serum (FBS), 2 mM L-glutamine, 5 mM HEPES, and 1 mM sodium pyruvate (all from Thermo Fisher Scientific, Waltham, MA, USA) (complete medium) in a 95% air–5% CO_2_ incubator at 37°C. For further selection, the cell suspension was incubated to allow pMø to adhere to the culture dish and then proceed with the analyses. RAW264.7 cells were maintained in DMEM (GIBCO, Thermo Fisher Scientific) with 10% FBS at 37°C in a humidified incubator containing 5% CO_2_. For stimulation, 1 or 0.5 × 10^6^ RAW264.7 cells or pMø, in complete medium with 0.5% FBS per well, were activated with the indicated stimuli and time points.

#### Bacterial Strains

Wild-type (*wt*) *Salmonella enterica* subsp. enterica serovar *typhimurium* (*S. typhimurium*, NCTC 12023) and attenuated, resistant to ampicillin, *S. enterica* subsp. enterica SseB^-^ (MvP643, p3232) strains were grown overnight under sterile conditions in LB medium (Sigma-Aldrich, St. Louis, MO, USA) and LB with 100 µg/ml of ampicillin (Sigma-Aldrich), respectively (31). Both the bacterial strains were kindly gifted by Dr. Michael Hensel, University of Osnabrück, Germany. *Escherichia coli* F18, *S. aureus*, and GFP-tagged bacteria have been previously described (4).

### Reagents

Antibodies against phospho-SHIP1 (Tyr1020), phospho-SHP-1 (Tyr564) (D11G5), phospho-Src family (Tyr416) (D49G4), phospho-p38 MAPK (Thr180/Tyr182) (3D7), phospho-(Ser) PKC substrate, phospho-p40^phox^ (Thr154), and Rac1/2/3 antibodies were purchased from Cell Signaling Technology. Phospho-ERK (E-4), p38α/β MAPK (H-147), p47^phox^ (H-195), p22^phox^ (C-17), Rab5 (D-11), Rab7 (H-50), calnexin (H-70), c-Myc (9E10), and COX-2 (C-20) antibodies were obtained from Santa Cruz Biotechnology (Dallas, TX, USA). The iNOS/NOS type II (54/iNOS) antibody was purchased from BD Biosciences, and the β-actin (AC-15) antibody was purchased from Sigma-Aldrich. Secondary antibodies for Western blotting, ECL™ anti-rabbit, and anti-mouse IgG HRP were obtained from GE Healthcare. For immunofluorescence and confocal laser microscopy, Alexa Fluor^®^488 goat anti-mouse IgG, Alexa Fluor^®^488 goat anti-rabbit IgG, Alexa Fluor^®^488 donkey anti-goat IgG, Alexa Fluor^®^594 goat anti-rabbit IgG, and Alexa Fluor^®^647 donkey anti-mouse IgG were obtained from Invitrogen. Phorbol 12-myristate 13-acetate (PMA) and pure lipopolysaccharide (LPS) from *E. coli* O111:B4 (L3024) were purchased from Sigma-Aldrich. The inhibitors, bisindolylmaleimide I (BIM-1), SB203580, and LY294992 were obtained from Calbiochem (San Diego, CA, USA).

### Cell Transfections

Overexpression of mouse SLAMF8 in RAW264.7 cells. Full-length mouse *Slamf8* cloned in pCMV6 (C-terminal Myc-DDK-tagged, MR203747) was purchased from Origene™ (Rockville, MD, USA). For stable transfection, 10^6^ RAW264.7 cells were electroporated with 1 µg of vector using Nucleofector™ Amaxa^®^(Cologne Nordrhein-Westfalen, Germany) (program D-032) according to the manufacturer’s instructions. Transient and stable *Slamf8*-RAW264.7 cells were cultured in complete medium with G-418. Then, *Slamf8* overexpression was analyzed by reverse transcription polymerase chain reaction (RT-PCR), Western blotting, and immunofluorescence. For Western blotting and immunofluorescence analyses, anti-c-Myc (9E10) antibody was used to detect the expression of the Myc-tag situated at the C-terminal position of mouse SLAMF8.

### Cell Treatments

Mø (1 or 0.5 × 10^6^per well) were stimulated with 100 ng/ml PMA or 10 µg/ml LPS at the indicated time points. For inhibition of kinases, Mø were pretreated with 5 μM BIM-1, 10 μM SB203580, or 10 μM LY294992 for 1 h and then activated with the indicated stimuli at the indicated time points. *In vitro infection model with S. typhimurium:* Mø were incubated with 100 U/ml IFNγ for 16 h prior to infection. Then, the cells were pulsed with wild-type (*wt*) *S. typhimurium* at a multiplicity of infection (MOI) of 10 and analyzed at different times post-infection. After pulse time, cells were washed with complete medium, supplemented with 10 μg/ml gentamicin (Sigma-Aldrich), and incubated for the corresponding times in order to kill extracellular bacteria. Subsequently, the cells were washed with cold PBS and lysed in appropriate lysis buffer to obtain the total extract.

### Western Blotting

For total extracts, after experimental treatments, Mø were washed with cold PBS and lysed in buffer containing 10 mM Tris–HCl (pH 7.6) (Sigma-Aldrich), 140 mM NaCl (Scharlau), 2 mM EDTA (Merck KGaA, Darmstadt, Germany), 1% NP40 (Thermo Scientific), 10 mM iodoacetamide, 5 mM sodium pyrophosphate, 1 mM sodium orthovanadate, 50 µM phenylarsine oxide, 50 mM sodium fluoride, 1 mM phenylmethylsulfonyl fluoride (all from Sigma-Aldrich), and 1× of protease inhibitor cocktail (Roche, Mannheim, Germany) for 30 min at 4°C.

#### Immunoblotting

All the extracts were resolved by SDS-PAGE, blotted, and probed with the indicated antibodies. Protein detection was carried out by chemiluminescence using ECL-Plus (Amersham, GE Healthcare) according to the manufacturer’s instructions, and protein bands were quantified by densitometry using Multi-Gauge software (V3.0, Fujifilm Life Science, Tokyo, Japan). The relative expression was normalized to the loading control.

#### Extraction of Cytosolic and Membrane Proteins

Mø were washed with cold PBS and lysed in buffer containing 100 mM HEPES (pH 7.3) (Sigma-Aldrich), 100 mM KCl (Panreac Qumica SLU, Barcelona, Spain), 3 mM NaCl, 3 mM MgCl_2_ (Sigma-Aldrich), 1.25 mM EGTA (Merck), 1 mM phenylmethylsulfonyl fluoride (Sigma-Aldrich), and 1× of protease inhibitor cocktail (Roche) for 30 min at 4°C. Then, the cells were sonicated, and non-lysed cells and debris were removed by centrifugation at 10,000 × *g* for 5 min at 4°C. Subsequently, the supernatants constituting the cytosolic fraction were further ultracentrifuged at 100,000 × *g* for 30 min at 4°C on an Optima™ MAX ultracentrifuge (Beckman Coulter, Pasadena, CA, USA) using a TLA-110 fixed angle rotor. The pellet was resuspended in a buffer containing 120 mM NaH_2_PO_4_ (pH 7.4) (Merck), 1 mM MgCl_2_ (Scharlau), 1 mM EGTA (Merck), 1 mM dithiothreitol, 20% (v/v) glycerol, 40 mM octylglucoside (all from Sigma-Aldrich), and 1× of protease and phosphatase inhibitor cocktail (Roche) and then recentrifuged at 20,000 × *g* for 40 min at 4°C to obtain the membrane fraction.

### Confocal Microscopy

#### Staining of F-Actin by Phalloidin-TRITC

Mø were plated on chamber slides (Thermo Scientific Nunc Lab-Tek). After experimental treatments, cells were washed with PBS and fixed with 2% paraformaldehyde (PDF) for 10 min at 4°C and subsequently with BD Cytofix/Cytoperm solution (BD Biosciences) for 20 min. After three washes with 0.05% PBS-saponin (Merck), cells were incubated with 5 μg/ml of phalloidin-TRITC (Sigma-Aldrich) in 0.05% PBS-saponin for 40 min at 4°C in the dark. Slides were washed twice with 0.02% PBS-saponin and then with PBS.

#### p47^phox^ and p22^phox^ Staining Analysis by Confocal Microscopy

pMø fixed with 2% PFD were washed three times with 0.1% PBS-Tween (Sigma-Aldrich) and incubated for 30 min on ice with 0.1% PBS-Tween20 containing purified rat anti-mouse CD16/CD32 antibody (BD Biosciences) at a dilution of 1:200 and 5% goat serum for p47^phox^ or 5% donkey serum for p22^phox^ staining, to block Fcγ receptors. After three washes with 0.1% PBS-Tween-20, the cells were incubated with α-p47^phox^ (H-195) or α-p22^phox^ (C-17) at a dilution of 1:100 in 0.1% PBS-Tween-20 containing 1% BSA. After 2 h of incubation, the cells were washed three times with 0.1% PBS-Tween-20 and incubated for 1 h in the dark with 0.1% PBS-Tween-20 containing 1% bovine serum albumin (BSA) and 1:1,000 dilution of Alexa Fluor^®^488 goat anti-rabbit IgG (Invitrogen, by Thermo Fisher Scientific) for p47^phox^ or Alexa Fluor^®^488 donkey anti-goat IgG (Invitrogen) for p22^phox^ staining. Slides were then washed twice with 0.1% PBS-Tween-20 followed by last wash with PBS. Finally, all the coverslips were mounted with DAPI-mounting medium (Vector Laboratories, Burlingame, CA, USA). Confocal microscopy was performed using an oil immersion 60× objective on the Nikon A1 microscope. The mean fluorescence intensity (MFI) was measured as the mean gray value of the maximum projection using ImageJ software.

### Phagocytic Uptake Analysis by Confocal Microscopy

pMø were plated on chamber slides (Nunc) and incubated with 100 U/ml IFNγ 16 h prior to infection. Then, the cells were infected at an MOI of 10 with *wt S. typhimurium* strain that expressed GFP at different pulse times. After experimental treatments, the cells were washed with PBS, fixed with 2% paraformaldehyde for 10 min at 4°C, and subsequently with BD Cytofix/Cytoperm solution (BD Biosciences) for 20 min. After three washes with 0.05% PBS-saponin (Merck), cells were incubated with 5 μg/ml of phalloidin-TRITC (Sigma-Aldrich) in 0.05% PBS-saponin for 40 min at 4°C in the dark. Slides were washed twice with 0.02% PBS-saponin followed by last wash with PBS. Finally, all the coverslips were mounted with DAPI-mounting medium (Vector Laboratories). Confocal microscopy was performed using a 60× objective on the Nikon A1 microscope. Orthogonal views were used to analyze the bacterial uptake, the percentage of macrophages infected, and the number of *S. typhimurium*-GFP per macrophage.

### Colocalization Analysis of *S. typhimurium-*GFP by Confocal Microscopy

pMø (1.5 × 10^5^ cells per well) were infected with wild-type *S. typhimurium*-GFP (MOI 10) at different pulse/chase times. The cells were then washed with complete medium supplemented with 10 μg/ml gentamicin (Sigma-Aldrich) to kill extracellular bacteria and incubated for the indicated chase times. After the experimental treatments, cells were washed and fixed as described above. After washing, pMø were incubated for 30 min with 0.1% PBS-Tween-20 containing 5% donkey serum (Sigma-Aldrich) and purified rat anti-mouse CD16/CD32 on ice to block Fcγ receptors.

#### Rab5 and Rab7 Staining

Cells were washed three times with 0.1% PBS-Tween-20 and incubated with α-Rab5 (D-11) and α-Rab7 (H-50) at a dilution of 1:100 in 0.1% PBS-Tween-20 containing 1% BSA for 2 h, and then washed and incubated with Alexa Fluor^®^647 donkey anti-mouse IgG (Invitrogen) and Alexa Fluor^®^594 goat anti-rabbit IgG (Invitrogen) at a dilution of 1:1,000 for 1 h in the dark.

#### p47^phox^ Staining

Washed cells were incubated with α-p47^phox^ (H-195) at a dilution of 1:100 in PBS-Tween-20 BSA for 2 h, washed three times, and incubated with Alexa Fluor^®^594 goat anti-rabbit IgG (Invitrogen) at a dilution of 1:1,000 in PBS-Tween-20 BSA for 1 h in the dark. All the slides were then washed twice with 0.1% PBS-Tween-20 followed by last wash with PBS. Finally, all the coverslips were mounted with DAPI-mounting medium (Vector Laboratories). Confocal microscopy was performed using a 60× objective on the Nikon A1 microscope and the colocalization was determined using the Pearson’s correlation coefficient of established regions of interest (ROIs) in z-stacks containing *S. typhimurium*-GFP and analyzed using NIS-Elements AR Analysis software (Nikon instrument Inc, Melville, NY, USA).

### *In Vivo* Infection With *S. typhimurium*

Mice were injected i.p. with *wt* and SseB^-^ *Salmonella* strains (5 × 10^4^ CFUs in 200 μl of PBS) and were sacrificed 48 h after infection. Peritoneal cells, obtained by peritoneal lavage, and spleen homogenates were lysed in appropriate lysis buffer to obtain total extracts. Then, the proteins were resolved by SDS-PAGE, blotted, probed with the respective antibodies, and analyzed as described above. For *in vivo* killing of *S. typhimurium*, mice were injected i.p. with the same amount of *wt S. typhimurium* and SseB^-^ (5 × 10^4^ CFUs in 200 μl of PBS) and were sacrificed 48 h after infection. Different dilutions of organ homogenates were cultured on LB plates with and without ampicillin (100 µg/ml) overnight at 37°C. The next day, total colony-forming units (CFUs) were calculated. The CFUs from *wt S. typhimurium* were calculated from the difference in CFUs in plates without ampicillin and CFUs in plates with ampicillin.

### Determination of ROS Production

pMø were plated in a 96-well plate and pretreated with or without the inhibitors, and 150 µM of lucigenin (Santa Cruz Biotechnology) in Hepes-buffered Krebs-Ringer (KR-Hepes), composed of 118 mM NaCl, 4.75 mM KCl, 1.18 mM H_2_PO_4_, 1.18 mM MgSO_4_, 1.25 mM CaCl_2_, 10 mM glucose, and 25 mM Hepes (pH 7.4) at 37°C for 1 h. Then, the cells were exposed to 100 ng/ml PMA, 10 µg/ml LPS, or *S. typhimurium* (MOI 10) and the chemiluminescence emission was measured at different times using the Synergy™ Neo2 hybrid multi-mode reader (BioTek, by Agilent, Santa Clara, CA, USA) and analyzed using the BioTek Gen5™ software. ROS production (NOX2 activity) was calculated as the increment of chemiluminescence over the value at t_0_ (before adding the activator agents).

### RT-PCR and Quantitative Reverse Transcription Polymerase Chain Reaction

After experimental treatments, total RNA was extracted with TRIzol reagent (Invitrogen) according to the manufacturer’s instructions. One microgram of RNA per sample was reverse transcribed into cDNA using the Reverse Transcription System (Promega, Madison, WI, USA) according to the manufacturer’s specifications. For overexpression of *Slamf8* in RAW264.7 cells, full-length mouse *Slamf8* primers were used (**Table 1**). The relative mRNA expression levels were determined by the 2^−ΔΔCt^ method, using the primer pairs for mouse as indicated in **Table 1**, by quantitative RT-PCR using FastStart SYBR Green Master mix (Roche) on the Quantica Real-Time PCR Thermal Cycler (Techne, Stone, Staffordshire, UK). Gene expression was normalized to the basal expression levels and graphically represented.

**Table 1:** Primers.

GEN	Forward	Reverse
TLR-4	GGGAGGCACATCTTCTGGAG	CCT CTG TTT GCT CAG
TLR-2	GGAGGTTGCATATCCCCCAG	GAG CAG GGA ACC AGG AAG AC
TLR-6	GTCAGTGGACACAGACCAGG	ACC CAG GCA GAA TCA TGC TC
SLAMF9	CCCATGAAGGCTCTGTCCTC	ACC CAG GCA GAA TCA TGC TC
SLAMF8-full length	ATGTGGTCCCTCTGGAGTCTTCTTC	CTATACGAGGGCATTCTCTGTCTCTGG
HTPRT1	CAACGGGGGACATAAAAGTTATTGGTGGA	TGACACTGGCAAAACAATGCA
SLAMF8 (RT-PCR)	ATGTGGTCCCTCTGGAGTCTTCTTC	GTACACCTTGGCTGGAGGGTC

### Determination of NO Production

NO production was determined by measuring the concentration of NO_2_^-^ in the culture medium using the Griess reaction. Upon complete activation, the supernatants were collected and centrifuged at 13,000 rpm for 5 min at 4°C in a microfuge to remove the cellular debris, and then incubated with the Griess reagent (1% sulfanilamide and 0.1% naphthylethylene-diaminedihydrochloride in 2.5% phosphoric acid) at room temperature for 30 min in the dark. The concentration of NO^-^ was calculated by comparison with the absorbance at 540 nm of standard solutions of 0–100 µM sodium nitrite in the appropriate culture media, using the Synergy™ Neo2 multi-mode reader (BioTek^®^) and analyzed using the BioTek Gen5™ software.

### Statistical Analysis

Statistical significance was determined by Student’s *t*-test (two-tailed distribution with a two-sample equal variance) and one-way ANOVA with Tukey’s post-hoc analysis. The *p*-values were considered significant when they were below 0.05.

## Results

### Enhanced Phosphorylation of NOX2 Subunits p47^phox^ and p40^phox^ *via* PKC, and MAPK ERK1/2 and p38 Activation in SLAMF8^-/-^ Macrophages

**Figure 1:**
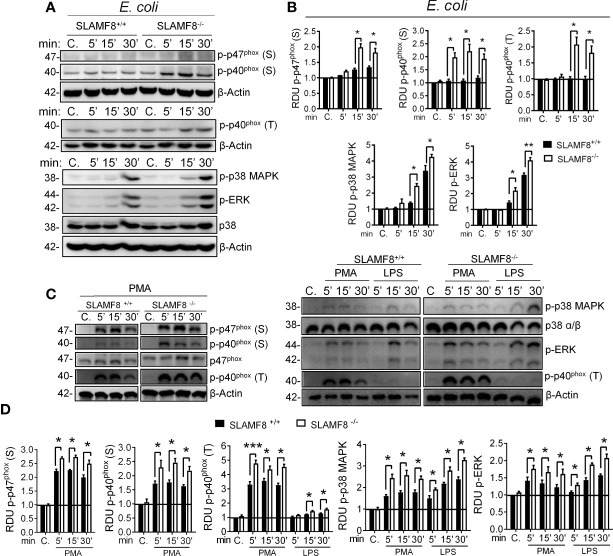
SLAMF8^-/-^ pMø show increased activation of NOX2 subunits and MAPK upon stimulation with bacteria or an agonist. Analysis of phosphorylated p47^phox^ and p40^phox^ NOX2 subunits, as well as ERK1/2 and p38 MAPK in *wt* and SLAMF8^-/-^ pMø upon stimulation with *E. coli* (MOI 10), 100 ng/ml PMA, or 10 µg/ml pure LPS at different time points. **(A, C)** Representative Western blots of the analyzed proteins in pMø upon stimulation with *E. coli*, PMA, or LPS. Results of one out of three independent experiments is shown. **(B, D)** Relative expression levels of phosphorylated proteins as indicated in A and C. Relative phosphorylation levels were normalized to appropriate loading control and are graphically represented. Values indicate mean ± SEM of three independent experiments. *p < 0.05; **p < 0.01; ***p < 0.001.* C*, control; S, serine; T, threonine; RDU, relative densitometry units.

We previously reported that SLAMF8*^-/-^* Mø showed increased ${\text{O}}_{2}{}_{-}^{•}$ production due to increased PKC activity and phosphorylation of threonine 154 (T154) in p40^phox^ upon bacterial and PMA, PKC agonist, stimulation (3, 4). Therefore, we postulated that SLAMF8 might modulate different NOX2 activation pathways (5). To test the hypothesis, primary peritoneal Mø (pMø) isolated from wild-type (*wt*) and SLAMF8^-/-^ mice were incubated with IFNγ and 0.5% FBS overnight, and then infected with *E. coli in vitro*. We analyzed the phosphorylation of ERK1/2 (p-pERK1/2) and p38 (p-p38) MAPK due to their involvement in NOX2 activation (14). In addition, the phosphorylation of p40^phox^ (p-p40^phox^) on T154 and the phospho-serine PKC substrate of subunits p47^phox^ (p-p47^phox^) and p40^phox^ were also analyzed by Western blotting (32). As expected, SLAMF8*^-/-^* pMø showed statistically significantly enhanced phosphorylation of the analyzed proteins compared to *wt* pMø (**Figures 1A, B**). We must emphasize that bone marrow-derived Mø or unprocessed pMø obtained by TGC showed a similar phenotype, so we used the latter in our assays (**Figure S1**) (4).

Next, to examine direct and indirect PKC activation, pMø were stimulated with PMA or pure LPS (<1% RNA and protein, Sigma-Aldrich) (22, 33, 34). SLAMF8^-/-^ pMø stimulated with PMA showed statistically significantly greater phosphorylation of p-p47^phox^ and p-p40^phox^ than *wt* pMø (**Figures 1C, D**). Stimulation with LPS showed statistically significantly greater phosphorylation of p-p40^phox^ (T) than *wt* pMø. Similarly, p-p38 and p-ERK1/2 MAPK were significantly increased in SLAMF8^-/-^ pMø compared to *wt* pMø, stimulated with either LPS or PMA (**Figures 1C, D**). Thus, SLAMF8 downmodulates not only PKC activation, but also the MAPK pathway. Considering these results, we hypothesized an increase in the mobilization of NOX2 cytosolic subunits toward the membrane in the absence of SLAMF8 in Mø, which was analyzed next.

### Enhanced Rac GTPase Mobilization and NOX2 Subunit Assembly to the Membrane in SLAMF8^-/-^ Macrophages

**Figure 2:**
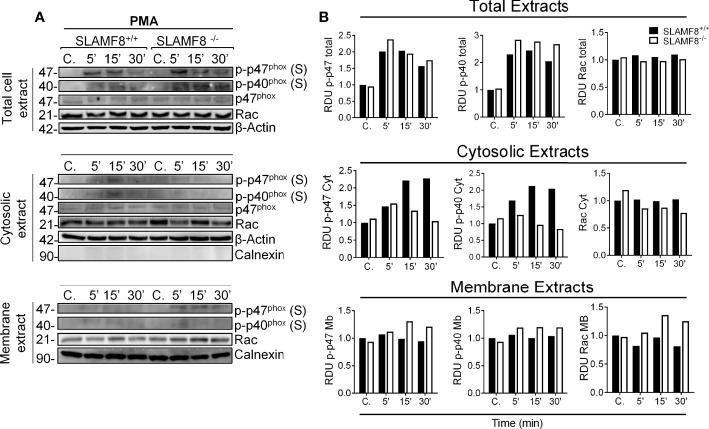
SLAMF8^-/-^ pMø show increased mobilization of cytosolic NOX2 subunits to the membrane. Analysis of Rac GTPase, p-p47^phox^, and p-p40^phox^ mobilization in SLAMF8^+/+^ and SLAMF8^-/-^ pMø stimulated with PMA (100 ng/ml). **(A)** Representative Western blots of total cell, cytosolic, and membrane extracts from SLAMF8^+/+^ and SLAMF8^-/-^ pMø treated with PMA at different time points. Absence and presence of Calnexin indicated purity of cytosolic and membrane extracts, respectively. **(B)** Relative expression levels of proteins indicated in A, representative data of one out of two independent experiments. C, control; S, serine; T, threonine; RDU, relative densitometry units.

**Figure 3:**
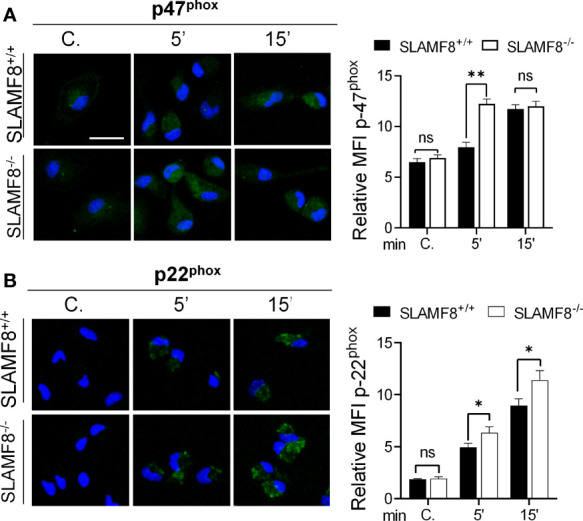
Increased fluorescence intensity staining of p47^phox^ and p22^phox^ in SLAMF8^-/-^ pMø indicates enhanced NOX subunit assembly compared to SLAMF8^-/-^ pMø. Immunofluorescence analysis of p47^phox^ and p22^phox^ in SLAMF8^+/+^ and SLAMF8^-/-^ pMø activated with PMA and stained with the indicated antibody. Representative images of pMø treated with PMA at different time points and stained with α-p47^phox^ **(A)** or α-p22^phox^ **(B)** (green). Cell nuclei were stained with DAPI, and the samples were analyzed by confocal microscopy. Scale bar: 20 µm. The mean fluorescence intensity (MFI) was measured as mean gray value (MFI) of maximum projection images and is graphically represented. Values indicate mean (maximum projection) ± SEM (*n* = 50 stochastic cells per coverslip). Results of one representative experiment out of two independent experiments are shown. *p < 0.05; **p < 0.001. C, control. ns, Not Significant.

To analyze the activation of the small Rho GTPases Rac, we performed Western blotting using cytosolic and membrane extracts from *wt* and SLAMF8^-/-^ pMø treated with PMA. Increased amounts of Rac GTPase subunit were observed in the cell membrane extracts of SLAMF8^-/-^ pMø compared to *wt* pMø (**Figure 2**). These differences were not due to the higher amount of total protein in SLAMF8^-/-^ pMø, since no differences were observed in whole protein extracts between the samples. On the other hand, cytosolic extracts show an inferior amount of the analyzed proteins in SLAMF8^-/-^ compared to *wt* pMø. An increased amount of p-p47^phox^ and p-p40^phox^ subunits was also observed in cell membrane extracts of SLAMF8^-/-^ pMø compared to their *wt* counterparts, which indicates a greater assembly of these two subunits at the cellular membrane compartments (32, 35) To verify this, we analyzed the MFI of anti-p47^phox^ and anti-p22^phox^ in pMø activated with PMA by confocal microscopy (**Figure 3**). These experiments showed higher MFI of p47^phox^ and p22^phox^ in SLAMF8^-/-^ than in *wt* pMø. Differences were statistically significant between SLAMF8^-/-^ and *wt* pMø (**Figures 3A, B**). All these data support the hypothesis that SLAMF8 modulates the PI3K and p38 MAPK, as Rac-GTP and NOX2 subunit assembly was reported to be regulated through these pathways (20, 25).

### SLAMF8^-/-^ Macrophages Show Increased PI3K-Dependent NOX2 Activation

**Figure 4:**
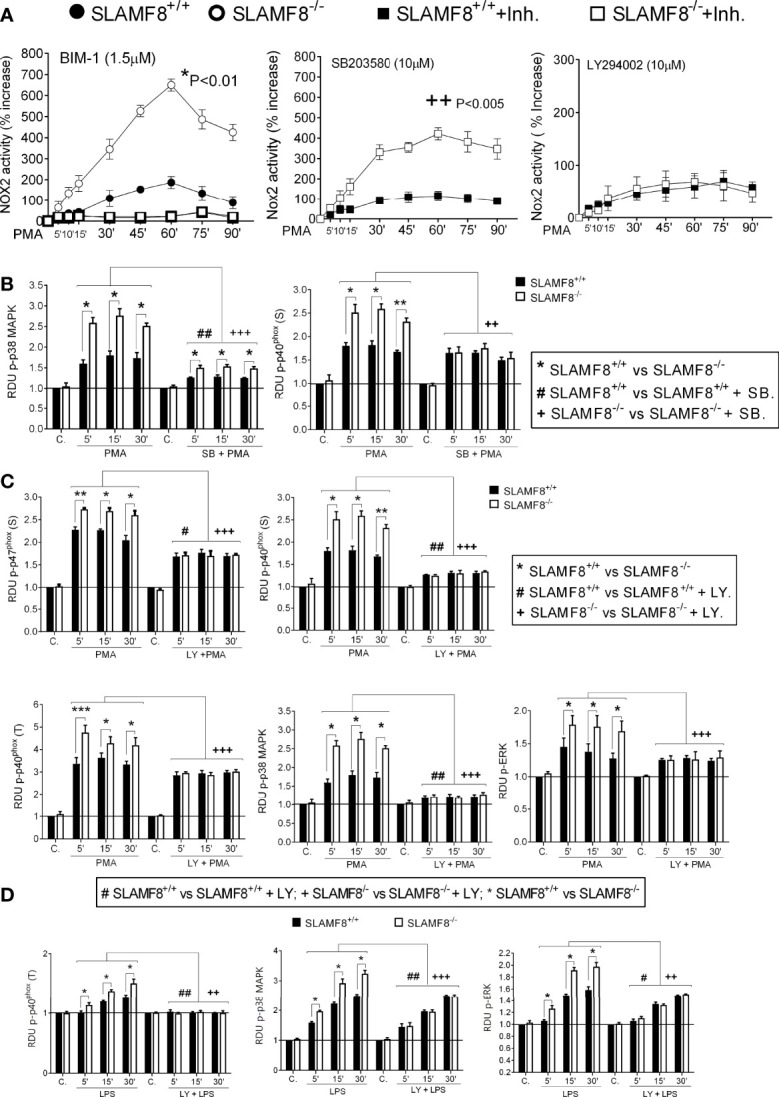
Study of ROS production and activation of NOX2 subunits in SLAMF8^+/+^ and SLAMF8^-/-^ pMø pretreated with inhibitors and stimulated with PMA or LPS. pMø were pretreated with or without 5 µM bisindolylmaleimide I (BIM1), 10 µM SB203580 (SB), or 10 µM LY294002 (Ly) for 1 h, and stimulated with 100 ng/ml PMA **(B, C)** or 10 µg/ml LPS **(C)** at different time points. **(A)** Analysis of ROS production monitored through lucigenin-luminescence assay, calculated as percentage increase over t_0_. Values indicate mean ± SEM. * Differences between SLAMF8^+/+^ pMø vs. SLAMF8^-/-^ pMø; + Differences between SLAMF8 ^+/+^ vs. SLAMF8^-/-^ samples treated with the indicated inhibitor. **(B–D)** The graphs show the relative expression levels of the indicated proteins, as analyzed by Western blotting (**Figures S2**, **S3**), in pMø stimulated with PMA **(B, C)** or LPS **(D)**. Levels were normalized to appropriate loading controls and are graphically represented. *Indicates differences between SLAMF8 ^+/+^ pMø vs. SLAMF8^-/-^ pMø at each time point, *p < 0.05; **p < 0.01; ***p < 0.001; ^#^ indicates differences between SLAMF8^+/+^ pMø vs. SLAMF8^+/+^ pMø treated with inhibitors. ^#^*p* < 0.05; ^##^*p* < 0.01; ^+^ indicates differences between SLAMF8^-/-^ pMø vs. SLAMF8^-/-^ pMø treated with inhibitors, ^++^*p* < 0.01; ^+++^*p* < 0.001. Values indicate mean ± SEM of three independent experiments. C, Control; RDU, relative densitometry units; S, Serine; T, threonine.

To consistently verify SLAMF8 downregulation in the different pathways, we analyzed ROS production and signal transduction in pMø treated with PMA, and pure LPS, to analyze PI3K activation, following exposure to specific inhibitors. For that, cells were pretreated with the PKC inhibitor bisindolylmaleimide I (BIM-1, 1.5 μM), selective p38 MAPK inhibitor SB203580 (10 μM), or the PI3K inhibitor LY294002 (10 μM) before stimulation as described (14, 24, 36, 37). Pretreatment with BIM-1 completely inhibited the production of *O*_2^−^_^•^ when pMø were activated with PMA in both types of cells (**Figure 4A**), and little or none of the phosphorylated proteins analyzed were observed (**Figure S2**). This result agrees with the broader inhibition of PKCs and other kinases with this inhibitor (38).

As previously described, pretreatment with the p38 MAPK inhibitor SB203580 significantly reduced ${\text{O}}_{2}{}_{-}^{•}$ production in PMA-activated pMϕ, although SLAMF8^-/-^ pMø showed a statistically significantly increased ${\text{O}}_{2}{}_{-}^{•}$ production compared to *wt* pMø (**Figure 4A**). Accordingly, the phosphorylation of p38 MAPK was reduced in both types of cells (19, 24), but continued to be statistically greater in SLAMF8^-/-^ pMø than in *wt* pMø at each time point of stimulation (**Figures 4B** and **S2**). Noticeably, the p-p40^phox^ on S was significantly reduced in SLAMF8^-/-^ pMø and became equivalent to the p-p40^phox^ on S in *wt* pMø (**Figures 4B** and **S2**), suggesting the SLAMF8 modulation of p38 MAPK activation. These results also suggested that p38 MAPK is directly involved in this subunit activation upon PMA treatment (not previously documented).

Activation of NOX2, dependent on the PI3K, was analyzed by pretreatment of cells with LY294002 (10 mM), which, as described (23, 36), reduced ROS production almost to basal levels (**Figure 4A**). Inhibition of PI3K not only reduced the phosphorylation of all the analyzed proteins in both types of cells, but unexpectedly decreased the observed differences between SLAMF8^-/-^ and *wt* pMø stimulated with PMA (**Figure 4C**). All these results highlight that intervention of SLAMF8 in the PI3K pathway modulates the activation of the majority of NOX2 subunits.

To confirm the increased activation of the PI3K pathway in SLAMF8^-/-^ pMø, we analyzed pure LPS stimulation in SLAMF8^-/-^ and *wt* pMø pretreated with the specific inhibitors as mentioned previously (39). Pretreatment with LY294002 showed reduced phosphorylation of p38 MAPK, ERK1/2, and p-p40^phox^ on T154 in both types of LPS-treated cells. No differences in the phosphorylated proteins analyzed were observed between SLAMF8^-/-^ and *wt* pMø (**Figures 4D** and **S3**). These results demonstrate that SLAMF8 negatively modulates NOX2 and Mø activation through the PI3K pathway.

### Increased NOX2 and iNOS Activation in *Salmonella*-infected SLAMF8^-/-^ Macrophages

**Figure 5:**
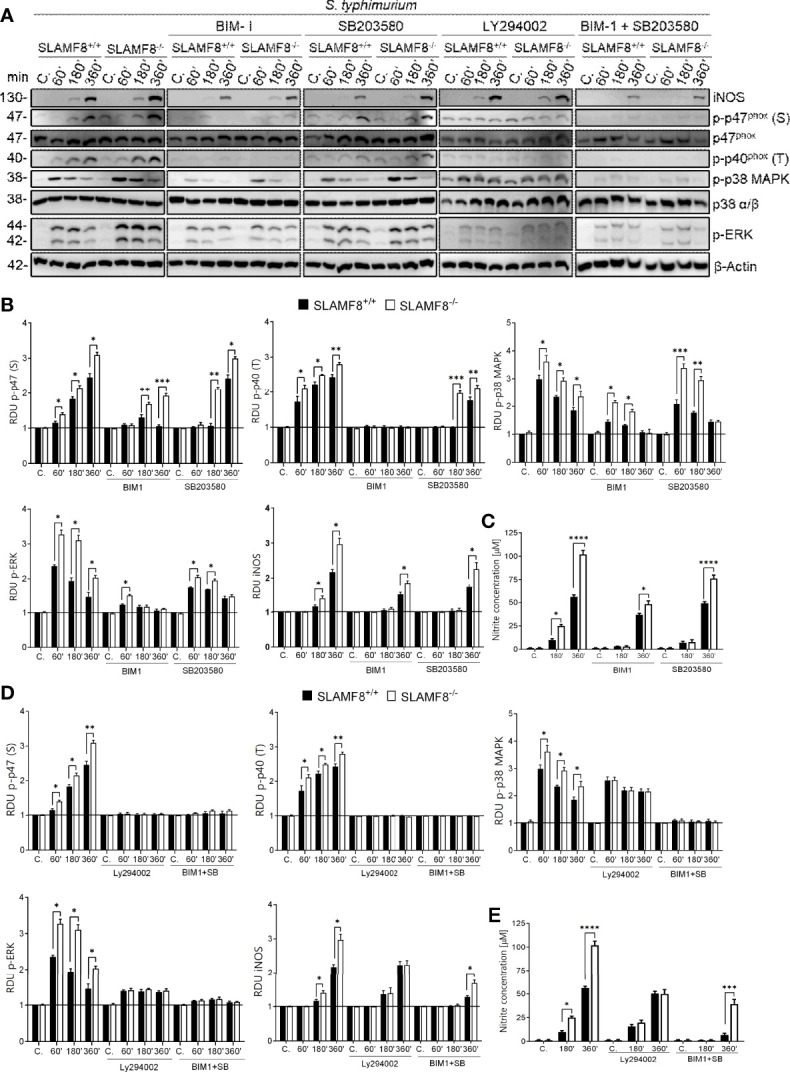
Analyses of activation of NOX2 subunits, ERK1/2, and p38 MAPK in SLAMF8*^+/+^* and SLAMF8^-^*^/-^* pMø upon infection *in vitro* with *S. typhimurium*, and determination of NO production. pMø were pretreated with or without 5 µM bisindolylmaleimide I (BIM1), 10 µM SB203580 (SB), or 10 µM LY294002 (Ly) for 1 h, and then infected with *S. typhimurium* (MOI 10) at different chase times. **(A)** Representative Western blots of indicated protein lysates. Results of one out of three independent experiments are shown. **(B, D)** Relative expression of phosphorylated proteins indicated in A; levels were normalized to appropriate loading controls and are graphically represented. *Indicates differences between SLAMF8 ^+/+^ vs. SLAMF8^-/-^ cells at each time point, *p < 0.05; **p < 0.01, ***p < 0.001. **(C, E)** Analysis of NO production in SLAMF8^+/+^ and SLAMF8^-^*^/-^* pMø infected with *S. typhimurium.* Histogram shows nitrite concentration [μM] as determined by the Griess reaction. Values indicate mean ± SEM. *Indicates differences between SLAMF8 ^+/+^ vs. SLAMF8^-/-^ cells at each time point, *p < 0.05; ***p < 0.001; ****p <0.0001. C, control; RDU, relative densitometry units; S, Serine; T, Threonine.

*S. enterica serovar typhimurium* (*wt S. typhimurium*) virulence is associated with its invasive capacity and suppression of the innate immune system. This bacterium is used as a model of typhi fibers and gastroenteritis *in vivo* and *in vitro* (26). Since IFNγ increases SLAMF8 expression (3, 4), and activation of pMø through IFNγ is crucial for *Salmonella* infection (40), we decided to analyze the impact of SLAMF8 in *Salmonella*-infected Mø. To emulate the infectious context, we treated pMø with IFNγ before *in vitro* stimulation. To follow *Salmonella*-containing vacuole (SCV) biogenesis, analyses *in vitro* were performed at early (<30 min post-infection), intermediate (30 min to 5 h), and late SCV stages (>5 h post-infection), at which point replication is initiated (41–43). In these experiments, pMø were pulsed for 15 min with *S. typhimurium*, and then samples were analyzed at the indicated times post-infection using the gentamicin protection assay (*Materials and Methods*). As observed with *E. coli*, SLAMF8^-/-^ pMø challenged with *S. typhimurium* (MOI 10) exhibited greater activation of NOX2 subunits, p-p40^phox^ on T154 and p-p47^phox^ on S, as well as phosphorylation of the kinases p38 MAPK and ERK1/2, than *wt* pMø (**Figures 5** and **S4**). The differences between samples were statistically significant. Note that, enhanced protein activation in SLAMF8^-/-^ pMø was not due to increased phagocytosis (**Figure S5**). We also performed analyses using specific inhibitors. Pretreatment of pMø with BIM-1 only partially reduced the phosphorylation of p47^phox^ (S), p38, and ERK1/2 MAPK, and inhibited the phosphorylation of p40^phox^ on T154 upon *S. typhimurium* stimulation, either in *wt* or in SLAMF8^-/-^ pMø (**Figures 5A, B**). Nonetheless, SLAMF8^-/-^ pMø still showed statistically significantly greater phosphorylation of all these proteins than *wt* pMø. We did not observe complete inhibition with BIM-1, possibly due to stimulation by several pathogen-associated molecular pattern receptors (PAMP-Rs) and pathways when Mø experienced whole bacterial stimulus, in contrast to chemical agonist treatment (**Figures S2**, **S3**). In fact, treatment with SB203580 partially reduced the phosphorylation of the analyzed proteins (**Figures 5A, B**), while the combined pretreatment with SB203580 and BIM-1 abolished NOX2 activation and signals in both types of cells (**Figures 5A, D**). Interestingly, as observed when using an agonist stimulus, pMø pretreatment with LY294002 and *S. typhimurium* showed reduced phosphorylation of the analyzed proteins and reduced the differences between SLAMF8^-/-^ and *wt* pMø (**Figures 5A, D**).

Inducible nitric oxide synthase (iNOS) is a key microbicidal mechanism involved in the clearance of *Salmonella* at later stages of infection and is dependent on phagolysosome formation (44, 45). Stimulation with *S. typhimurium* showed greater induction of iNOS (**Figures 5A, B**) and therefore a higher production of NO in SLAMF8^-/-^ pMø than in *wt* pMø (**Figure 5C**). Treatment with the PI3K inhibitor eliminated the differences between samples, either in iNOS expression (**Figures 5A, B**) or in the production of NO (**Figure 5D**). Pretreatment with BIM-1 and or SB203580 partially reduced iNOS and NO production, although there were still differences between SLAMF8^-/-^ and *wt* pMø (**Figures 5C, E**). These results confirmed the relevance of SLAMF8 in PI3K pathway activation in mouse Mø during *S. typhimurium* infection. Given these results, we analyzed whether SCV progression in SLAMF8^-/-^ pMø was impaired.

### Enhanced Recruitment of the Small GTPases Rab5 and Rab7, as Well as the p47^phox^ Subunit to *Salmonella* in SLAMF8^-/-^ Macrophages

**Figure 6:**
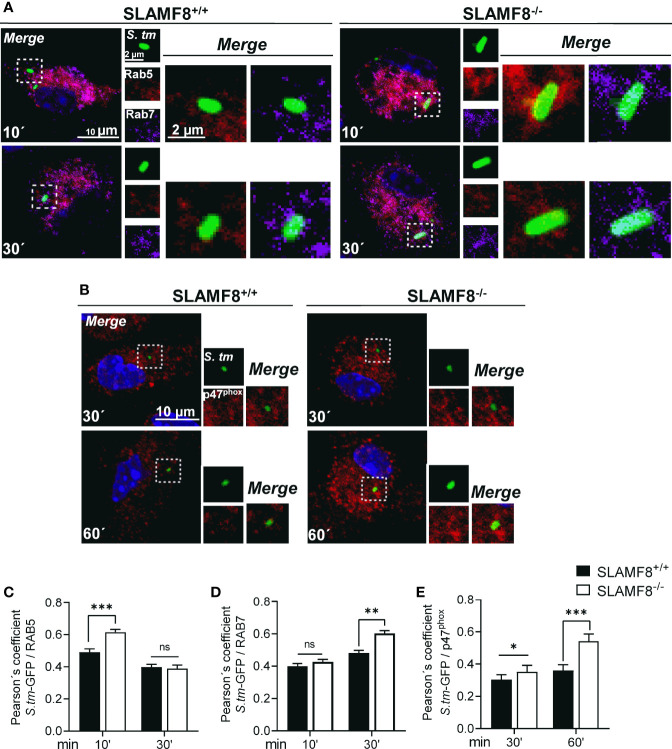
SLAMF8^-/-^ pMϕ show increased transport of Rab5, Rab7, and p47^phox^ to SCV compartment compared to SLAMF8^+/+^ pMø. Analysis of pMø infected with wild-type *S. typhimurium*-GFP (MOI 10) at different chase times and stained with antibodies against Rab5 (red), Rab7 (purple), and p47^phox^ (red). Nuclei were stained with DAPI. Representative confocal images of high-magnification views from z-stacks (merge) and maximum projection of each parameter (small square pictures) are shown. Scale bars are indicated. Colocalization of proteins with *S. typhimurium*-GFP was determined by the Pearson’s correlation coefficient. Analyses were performed using the Nikon software. **(A)** Representative pictures of Rab5 (red) and Rab7 (purple) are shown **(C, D)**. Graphs show colocalization of Rab5 and Rab7 with *S. typhimurium*-GFP in SLAMF8^+/+^ and SLAMF8^-/-^ pMø. *Indicates statistical significance between samples (*p<0.05, **p < 0.01, ***p < 0.001). **(B)** Representative pictures of p47^phox^ (red) and *S. typhimurium*-GFP in SLAMF8^+/+^ and SLAMF8^-/-^ pMø **(E)**. Colocalization of p47^phox^ (red) and *S. typhimurium*-GFP in SLAMF8^+/+^ and SLAMF8^-/-^ pMø. *Indicates statistical significance for p47 ^phox^ between samples (***p < 0.001). Values in the graph indicate the Pearson´s coefficient ± SEM (*n* = 50 stochastic cells per coverslip). Results of one out of three independent experiments are shown. ns, not Significant.

The maturation of SCV involves a continuous and dynamic interaction with the host endosomal system (46, 47). Given the previous results, we postulated that SLAMF8 might modulate SCV maturation and progression. To examine the levels of maturation markers in SLAMF8^-/-^ Mø compared to *wt* Mø, we analyzed the early to intermediate stages of SCV progression by confocal microscopy. Early SCVs (<30 min) were determined by the presence of the small GTPase Rab5 (47), and late SCVs were determined with GTPase Rab7 (>30 min) (48). Colocalization of GFP-*Salmonella* with these proteins was analyzed in z-stacks and calculated using Pearson’s correlation coefficient (41, 49). As shown in **Figure 6**, we observed significantly increased recruitment of Rab5 or Rab7 towards green-*Salmonella* in SLAMF8^-/-^ pMø compared to *wt* pMø. This observation was not due to greater phagocytosis, as evaluated by confocal microscopy, and there were no differences between *wt* and SLAMF8^-/-^ pMø samples, neither in the percentage of Mø infected, nor in the percentage of Mø with different number of *S. typhimurium* per cell (**Figures S5B, C**). These results suggested that in the absence of SLAMF8, the Mø exhibit increased phagosome–lysosome fusion (50).

On the other hand, to corroborate greater activation of NOX2 upon SCV, we analyzed p47^phox^ localization in GFP-*Salmonella* in pMø by confocal microscopy (35). Analyses were also performed in z-stacks and using Pearson’s correlation coefficient. We observed statistically significantly enhanced approximation of p47^phox^ to GFP-*Salmonella* in SLAMF8^-/-^ pMø compared to *wt* pMø (**Figures 6B, E**). This result corroborated the increase in NOX2 activation over *Salmonella*, and suggests an increased SCV progression in SLAMF8^-/-^ pMø compared to *wt*, similar to the results observed upon stimulation with agonist PMA.

### Activation of SLAMF8^-/-^ Macrophages With IFNγ and *S. typhimurium* Shows Increased Expression of IL-6 Production, But No Differences in TLRs or SLAMF9

**Figure 7:**
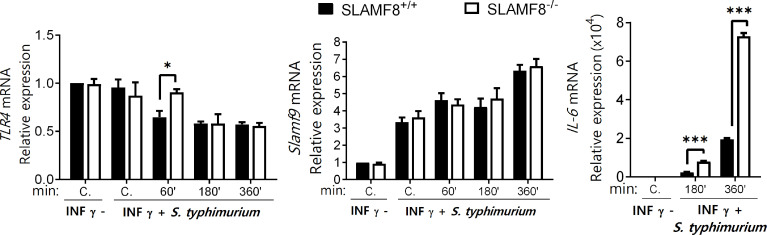
SLAMF8^-/-^ pMø show differences in IL-6 expression, but not in SLAMF9 or TLRs expression. Expression levels of the indicated mRNAs as determined by quantitative RT-PCR in pMø treated with or without IFNγ (100 U/ml) for 16 h, and then infected with *wt S. typhimurium* (MOI 10) at different chase times. Expression of gene expression was normalized to basal expression levels of untreated cells (0/0). Data were analyzed by the 2^−ΔΔCt^ method and *HPRT1* was used as the reference gene. Results of two independent experiments are shown. *p < 0.05; ***p < 0.001. C, Control.

Recently, Zeng et al. (51) showed that the combined interaction of SLAMF9 and SLAMF8 modulates the response to LPS through TLR4 receptor in mice. To evaluate whether SLAMF8 can modulate the expression of TLR4 and SLAMF9 and, therefore, influence the observed phenotype in SLAMF8-deficient mice, we analyzed their expression in pMø by RT-PCR after treatment with IFNγ (100 U/ml for 16 h) and infection with *S. typhimurium* at different time points (**Figure 7**). The expression of *tlr4* was only significantly greater at a pulse chase of 15/60 min post-stimulus in SLAMF8^-/-^ pMø compared to *wt* pMø, whereas no differences were observed in the expression of *Slamf9* during the experimental time points. Analyses of *tlr2* and *tlr6* showed similar results (**Figure S6**). On the other hand, and in consonance with increased PI3K signal, the IL-6 expression in pMø stimulated with IFNγ plus *Salmonella in vitro* was higher in SLAMF8^-/-^ pMø than in *wt* pMø (**Figure 7**). These results indicate that signal transduction and inflammatory response during *S. typhimurium* infection are modulated by SLAMF8 in mouse Mø.

### SLAMF8^-/-^ Macrophages Show Increased Src Kinase Activation and Reduced SHP-1 Phosphorylation Upon IFNγ and *Salmonella* Stimuli

**Figure 8:**
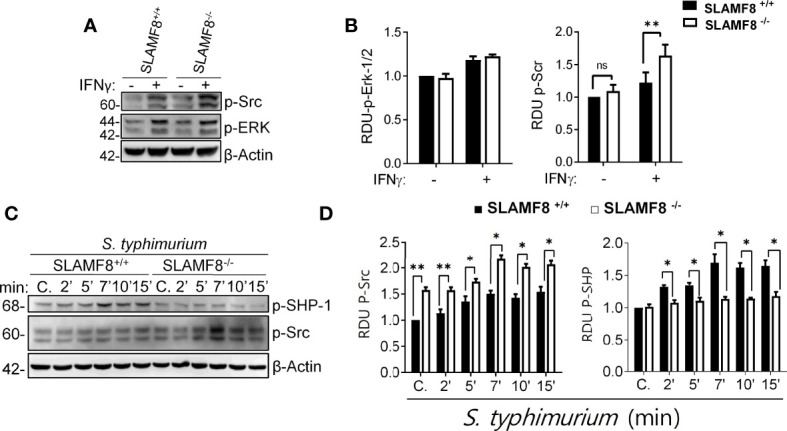
SLAMF8 controls phosphorylation of Src and SHP-1 in pMø upon *in vitro S. typhimurium* infection. **(A, C)** Representative Western blots (left) of the indicated proteins in pMø, **(A, B)** treated with or without IFNγ (100 U/ml) for 16 h, and **(C, D)** treated with IFNγ with or without *wt S. typhimurium* (MOI 10) at different chase times. **(B, D)** Graphs represent the relative expression of the indicated proteins (right). Values indicate mean ± SEM of three independent experiments *p < 0.05, **p < 0.01. C, Control; RDU, relative densitometry units; S, Serine; T, Threonine.

The activation of innate cells occurs through distinct Src kinases, which are also defined as integrators of different signaling pathways in Mø that experience an external stimulus (52). *Salmonella* infection has been shown to induce Src kinase activation during internalization (53). Therefore, we analyzed Src kinase activation in resting and treated pMø. Evaluation of Src kinase activity showed a statistically significant increase in the phosphorylation of Tyr416 in SLAMF8^-/-^ pMø compared to *wt* pMø treated with IFNγ (16 h) *in vitro* (**Figures 8A, B**). However, there were no differences in the phosphorylation levels of the other proteins analyzed, e.g., Erk1/2 kinase (**Figures 8A, B**) and others (**Figure S7**). Exposure to *S. typhimurium* significantly increased the differences in the phosphorylation of Src on Tyr416 between SLAMF8^-/-^ and *wt* pMø (**Figures 8C, D**). Alternatively, cytosolic protein tyrosine phosphatases (PTPs) have been defined as keepers of Mø activation, and SHP-1 has been specifically implicated in the negative regulation of superoxide production by NOX2 through the PI3K-Rac GTPase signaling pathway (54, 55). Statistically significantly decreased phosphorylation of SHP-1 on Tyr564 was detected in SLAMF8^-/-^ pMø compared to *wt* pMø treated with IFNγ and *S. typhimurium* (**Figures 8C, D**).

### Overexpression of SLAMF8 Confirmed Its Negative Modulation of Mouse Macrophage Activation

**Figure 9:**
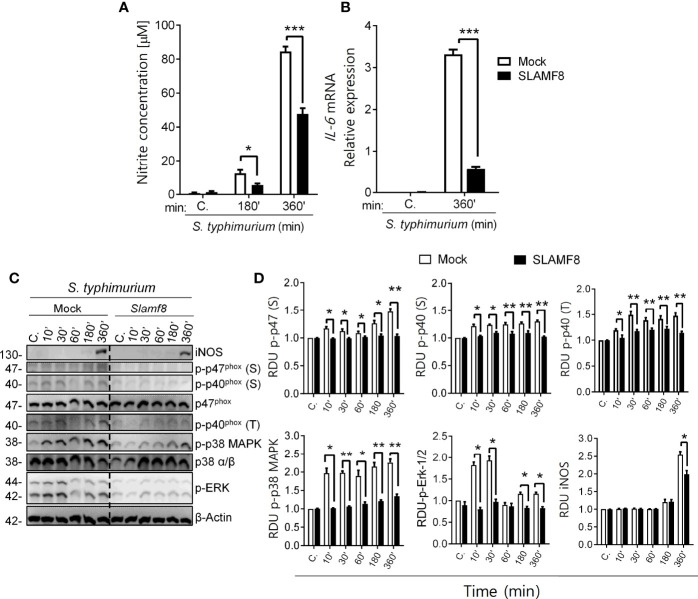
Study of NOS type II induction, NOX2 activation through phosphorylation analysis of p47^phox^, p40^phox^, and p38/ERK MAPK in *SLAMF8* or mock-transfected RAW264.7 Mø. Determination of NO production and *IL-6* mRNA expression. Stable clones isolated from RAW264.7 Mø transfected with cDNA encoding Myc-tagged *Slamf8* or mock-transfected cells were infected with *S. typhimurium* (MOI 10) at different chase times. The protein samples were subjected to Western blot analysis and results of one out of three independent experiments are shown. **(A)** Analysis of nitrite production in *Slamf8* or mock-transfected RAW264.7 Mø. Histogram shows nitrite concentration [μM], as determined by the Griess reaction, in Mø infected with *S. typhimurium* at different chase times. Values indicate mean ± SEM. **(B)** Relative expression of *il-6* mRNA in Myc-tagged *Slamf8* or mock-transfected RAW264.7 cells infected with *S. typhimurium* (MOI10) as analyzed by quantitative RT-PCR. **(C)** Representative Western blots of indicated phosphorylated proteins and levels of iNOS/NOS type II are shown. **(D)** Relative expression of phosphorylated proteins as indicated in **(C)** Phosphorylation levels were normalized to appropriate loading control and are graphically represented. Gene expression was normalized to basal expression levels of mock-transfected RAW264.7 cells. Data were analyzed by the 2^−ΔΔCt^ method, and *HPRT1* was used as the reference gene. *p < 0.05; **p < 0.01; ***p < 0.001. C, control; MOI, multiplicity of infection; RDU, relative densitometry units; S, Serine; T, Threonine.

Finally, to confirm the role of SLAMF8 in mouse macrophages, we analyzed whether overexpression of SLAMF8 showed the opposite phenotype. RAW264.7 Mø transfected with cDNA encoding Myc-tagged *Slamf8* were infected with wild-type *S. typhimurium* (MOI 10), and analyses were performed at different follow-up times. Stable overexpression of *Slamf8* in RAW264.7 cells reversed SLAMF8^-/–^associated phenotype. Hence, the relative expression levels of iNOS and IL-6 were lower in *Slamf8-*transfected RAW264.7 cells compared to mock-transfected cells when stimulated with *S*. *typhimurium* (**Figures 9A, B**). Overexpression of *Slamf8* also showed statistically significantly lower phosphorylation of p40*^phox^* on S and T154, and p47*^phox^* on S, p38 MAPK, and ERK1/2 compared to mock-transfected RAW264.7 cells (**Figures 9C, D**). In accordance, the same results were observed in RAW264.7 cells stimulated with PMA and LPS (**Figures S8**, **S9**). These results corroborate SLAMF8-mediated negative modulation of NOX2 in mouse Mø during *S. typhimurium* infection. Given all the *in vitro* results in the absence of SLAMF8, we analyzed *Salmonella* clearance *in vivo*.

### SLAMF8^-/-^ Mice Exhibit Greater *S. typhimurium* Clearance *In Vivo*

**Figure 10:**
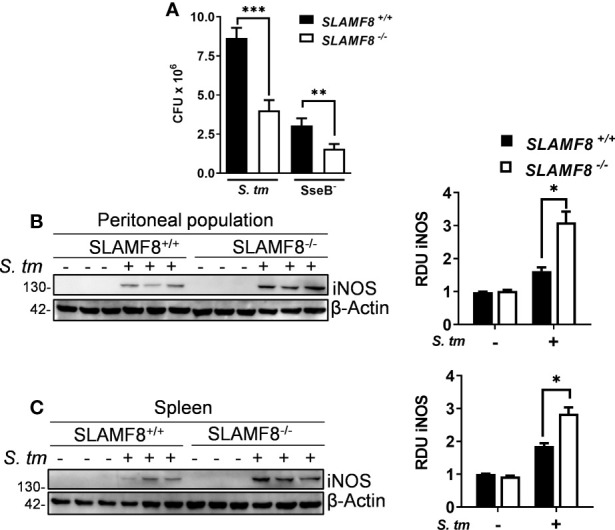
SLAMF8^-/-^ mice exhibit greater bactericidal activity and NOS induction upon *S. typhimurium* infection *in vivo*. **(A)** Analysis of bactericidal activity in SLAMF8^+/+^ and SLAMF8^-/-^ mice. Mice were injected i.p. with wild-type *S.* typhimurium and attenuated SseB^-^ strains (5 × 10^4^ CFUs) and subsequently sacrificed, and serial dilutions of spleen homogenates were plated on LB agar plates with and without ampicillin (100 ng/ml). Values indicate mean ± SEM of triplicate dilutions from one out of three independent experiments (*n* = 5 mice per experiment). **(B, C)** Analysis of microbicidal mechanisms in SLAMF8^+/+^ and SLAMF8^-/-^ mice. Mice were infected with *S. typhimurium* (5 × 10^4^ CFUs) or uninfected (PBS). **(B)** Peritoneal population and **(C)** spleen lysates from three individual mice were obtained, after 48 h of i.p. injection, and subjected to Western blotting for the indicated proteins (left). Relative expression levels were normalized using β-actin as the loading control, and graphically represented (right). Values indicate mean ± SEM of three individual mice used in one out of two experiments. *p < 0.05; **p < 0.01; ***p < 0.001. RDU, relative densitometry units; CFU, colony-forming units.

Since SLAMF8^-/-^ Mø showed increased iNOS, and NOX2 activity *in vitro*, we wondered whether SLAMF8^-/-^ mice develop an altered microbicidal response following *S. typhimurium* infection compared to *wt* mice. To analyze bacterial clearance, we used two strains of *Salmonella*: *wt S. typhimurium*, which could not be managed by mice (56), and the attenuated *S. typhimurium* SseB^-^ to evaluate microbicidal ability (31, 56). Wild-type and SLAMF8^-/-^ mice were injected intraperitoneally (i.p.) with the same amount of *Salmonella wt* and SseB^-^ bacteria [5 × 10^4^ colony-forming units (CFUs)], and the number of CFUs was evaluated in tissue homogenates 48 h post infection. As expected, the bacterial burden of *wt Salmonella* and SseB^-^ isolated from spleen homogenates of SLAMF8^-/-^ mice was significantly lower than that in *wt* mice (**Figure 10A**). Moreover, the incremental number of *wt Salmonella* CFUs obtained from *wt* mice compared to that in SLAMF8^-/-^ mice indicated reduced replicative capability of *wt Salmonella* in SLAMF8-deficient mice (31, 49). Hence, SLAMF8^-/-^ mice exhibited significantly greater bactericidal activity and infection control than *wt* mice. Consistently, analysis of mice infected with *S. typhimurium* also demonstrated significantly increased NOS induction in SLAMF8^-/-^ mice compared to *wt* mice, either in the peritoneal population or in spleen homogenates (**Figures 10B, C**).

## Discussion

In this study, we demonstrate the role of SLAMF8 in the negative modulation of Mø activation *via* the PI3K pathway and its impact on the SCV progression and clearance of *Salmonella*. We believe that SLAMF8 downregulates the PI3K-PKC positive-feedback loop, since specific inhibition using LY294002 eliminated the differences observed between SLAMF8^-/-^ and *wt* pMø, upon stimulating with either bacteria or PKC agonist. This agrees with the previous observation of increased polarization and migration of SLAMF8^-/-^ pMø compared to *wt* pMø (3), which is regulated through the PI3K-PKC positive-feedback loop (57, 58). On the other hand, PI3K inhibition also reduced the differences in MAPK activation, which is also dependent on PI3K (22), while with the p38 MAPK inhibitor, SB203580, significant differences were still observed between the two types of cells. This result indicates a p38 MAPK positive feedback on NOX2 subunit activation. Furthermore, our results indicate that SLAMF8 negatively modulates primary early signaling in Mø, since priming SLAMF8^-/-^ Mø with IFNγ or pure LPS showed increased Src and PI3K activation, respectively, compared to *wt* Mø. The activation of Src kinase through IFNγ signaling has been documented in human and mouse Mø (59–61), as well as the participation of Src kinase upon PI3K-dependent NOX2 activation in *Salmonella* or LPS-stimulated Mø (53, 62). Additionally, increased ROS production enhanced the activation of Src kinases and reduced SHP-1 activity (63, 64) in the absence of SLAMF8, thereby promoting further increase in cell activation. In fact, inhibition of ROS with diphenyleneiodonium was documented to reduce the enhanced migration of SLAMF8^-/-^ Mø [4].

Regulation of NOX2 activation and phagosome–lysosome fusion are key mechanisms to ensure *Salmonella* survival and replication in infected Mø (55, 65). In agreement with SLAMF8-mediated negative modulation of PI3K signal and phagosome NOX2 activity, we observed impairment of *Salmonella* inhibition during early SCV progression determined by increased p47^phox^ recruitment in SLAMF8^-/-^ pMø (35, 47, 48), and resulted in increased NO production (66). All these are in consonance with the inferior phagosome acidification in SLAMF8^-/-^ Mø (4), and thus reduced *Salmonella* replication and greater bacterial clearance in SLAMF8^-/-^ mice. In fact, acidic pH of the SCV favors *Salmonella* survival and replication (67). Therefore, the strategy of *Salmonella* to inhibit early SCV progression is impaired in SLAMF8-deficient Mø (48). The mechanism underlying this phenomenon requires further analysis. Nonetheless, these results suggest that SLAMF8 expression may also modulate phagosome–lysosome fusion mechanisms in mouse Mø. Modulation of phagosome microbicidal mechanisms by SLAMF1 has also been documented (50), but in contrast to SLAMF8, SLAMF1-deficient pMø showed delayed phagosome maturation and lower phagosomal pH (50). These data suggest that SLAMF8 may adjust the phagosome by downregulating SLAMF1 signaling. The molecular mechanism that mediates SLAMF8 inhibition is unraveled. Since SLAMF8 is itself ligand and has no known cytoplasmic motifs, it is unlikely that it operates signal inhibition through any adaptor protein. One possibility would be that SLAMF8 functions in competitive inhibition with SLAMF1 (2). In this sense, SLAMF8 interaction with itself in the same cell (*cis* interaction) may reduce SLAMF1 interaction with either neighboring cells or the pathogen (*trans* interaction), as described for SLAMF2 in NK cells (68).

Recently, Zeng et al. (51) proposed the combined role of SLAMF8 and SLAMF9 in response to LPS-mediated sepsis, downregulating TLR4 in Mø. As mentioned, pure LPS treatment increased the priming of NOX2 in SLAMF8^-/-^ pMø compared to *wt* pMϕ (69, 70). The authors did not observe any phenotype in single knock out RAW264.7 Mø for SLAMF8 or SLAMF9. In contrast, we did not observe differences in TLR expression in *Salmonella*-infected SLAMF8-deficient pMø compared to wt (**Figure S6**), but we described increased IL-6 expression compared to *wt* pMø, which might also enhance Mø activation. Although our study analyzed the *Salmonella* sepsis model, which is a cytoplasmic germ, and used different techniques, we believe that SLAMF9, in addition to SLAMF1, may function to increase Mø activation in the absence of SLAMF8. Noticeably, other authors have shown a detrimental effect in plasmocytic dendritic cell functionality and in *Salmonella*-infected mice dependent on single SLAMF9 deficiency (71, 72). On the other hand, we should not exclude the possibility, as shown for other SLAMF members, that SLAMF8 can operate as a PAMP-R. The modulation of Mø activation by SLAMF receptors in association with TLRs has also been described (2). For instance, SLAMF1 mediates TLR4 signaling by forming a complex with PI3K-Vps34, both in humans and in mouse Mø (73, 74).

In conclusion, early intervention of SLAMF8 upon bacterial encounter modulates early IFNγ and PAMP-R signaling dependent on PI3K pathway, reducing microbicidal activation of mouse Mø. All these data suggest that SLAMF8 could be a target for therapeutic intervention in the control of chronic or inflammatory immune responses.

## Data Availability Statement

The original contributions presented in the study are included in the article/**Supplementary Material**. Further inquiries can be directed to the corresponding author.

## Ethics Statement

The animal study was reviewed and approved by the Ethics Committee of Animal Experimentation, University of Granada (References: CEEA-379 y CEEA-417-2012). Written informed consent was obtained from the owners for the participation of their animals in this study.

## Author Contributions

SR-P, DR-B, LM-d-L, and ACA-M performed research and the statistical analysis of the results. D-RB and MR-M were involved in data collection and analysis and assisted in performing the experiments. EM-G assisted in performing RAB5 and RAB7 experiments. FA-M and CT contributed to the assembly and assisted in performing the experiments. ACA-M designed the different experiments, interpreted the results, contributed to the financial support, and wrote the manuscript. SR-P and MR-M helped to edit and reviewed the manuscript. All authors contributed to the article and approved the submitted version.

## Funding

This research project was supported by the *Plan Estatal de Investigación Científica y Técnica y de Innovación*, *ISCIII-Subdirección General de Evaluación y Fomento de la Investigación, Ministerio de Economía y Competitividad*, Spain (Grants PI16/01642 and PI10/01096).

## Conflict of Interest

The authors declare that the research was conducted in the absence of any commercial or financial relationships that could be construed as a potential conflict of interest.

## Publisher’s Note

All claims expressed in this article are solely those of the authors and do not necessarily represent those of their affiliated organizations, or those of the publisher, the editors and the reviewers. Any product that may be evaluated in this article, or claim that may be made by its manufacturer, is not guaranteed or endorsed by the publisher.

## Abbreviations

CFU, colony-forming units; iNOS, inducible nitric oxide synthase; Mø, macrophages; MOI, multiplicity of infection; NADPH, nicotinamide adenine dinucleotide phosphate oxidase; NOX2, NADPH oxidase; ${\text{O}}_{2}{}_{-}^{•}$ , superoxide anion radical; pMϕ, peritoneal macrophages; MIF, mean intensity of fluorescence; PAMP-R, pathogen-associated molecular pattern; ROS, reactive oxygen species; SCV, *Salmonella*-containing vacuole; *S. typhimurium*: *Salmonella typhimurium*; S, serine; T, threonine; to, time; Tyr, tyrosine; wt, wild type.
