# Supplementary material for: SLAMF8 Downregulates Mouse Macrophage Microbicidal Mechanisms via PI3K Pathways
Source: Front Immunol. 2022 Jun 28;13:910112. doi: 10.3389/fimmu.2022.910112 (PMC9273976; doi:10.3389/fimmu.2022.910112)
Supplement: Supplementary file 1 [file DataSheet_1.pdf]

SUPLEMENTARY FIGURE

Figure S1

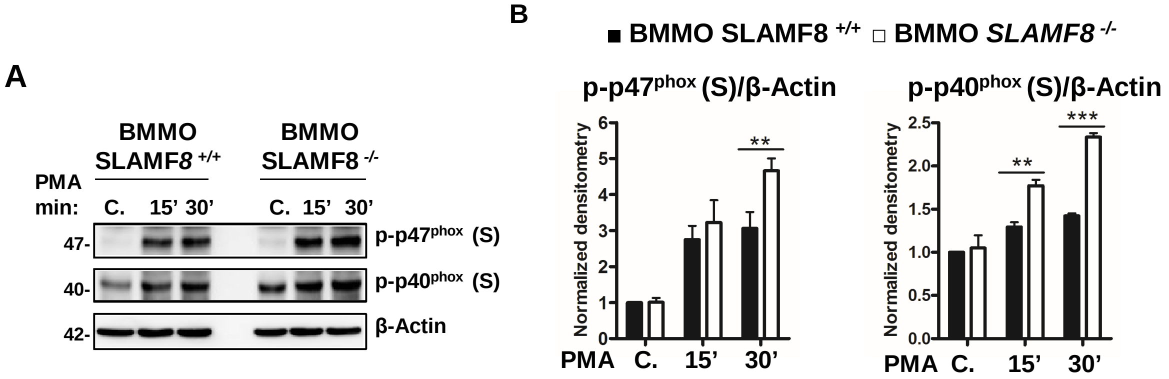

S1 Figure. Bone marrow-derived macrophages of **SLAMF8$^{-/-}$** ***mice show increased NOX2-subunit activation upon PMA agonist stimulation.*** Analysis of phosphorylated p47^{phox} and p40^{phox} in SLAMF8$^{+/+}$ and SLAMF8$^{-/-}$ bone marrow derived Mø stimulated with PMA [100 ng/ml] at different time points. **(A)** Representative western blots of analyzed proteins in Mø stimulated with PMA. Results of one out of three independent experiments is shown. **(B)** Relative expressions levels of phosphorylated proteins as indicated in A. Relative phosphorylation levels were normalized to appropriate loading control and are graphically represented. Values indicate mean ± SEM of three independent experiments. *p<0.05; **p<0.01. C., control; R, resting cells; S serine; T, threonine; RDU, relative densitometry units. BMMO: bone marrow derived macrophages. S, Serine; T, Threonine.

Figure S2.

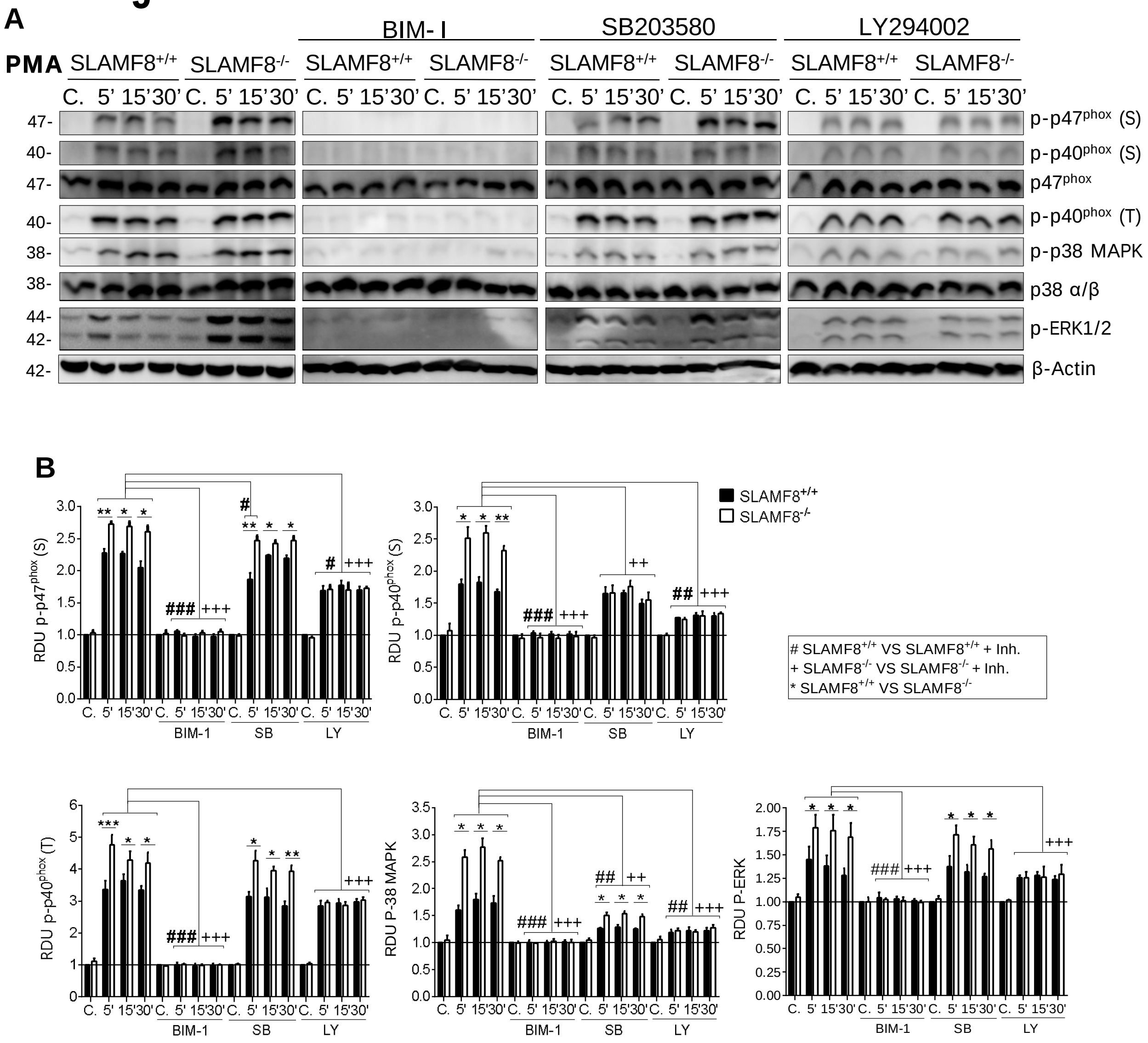

Figure S2. Study of NOX2 activation through phosphorylation analysis of p47^{phox}, p40^{phox}, p38/ERK MAPK in SLAMF8$^{-/-}$ pretreated with inhibitors and stimulated with PMA. pMø were pretreated with or without 5 µM bisindolylmaleimide I (BIM-1), 10 µM SB203580 (SB), or 10 µM LY294002 (Ly) for 1 h, and stimulated with 100 ng/ml of PMA at different time points. **(A)** Representative western blots of the indicated proteins in pMø pretreated with or without the indicated inhibitors and stimulated PMA. Results of one out of three independent experiments are shown. (**B)** Relative expression levels of phosphorylated proteins as indicated in A. Levels were normalized to appropriate loading controls and are graphically represented. *****Indicates differences between SLAMF8 $^{+/+}$ pMø vs SLAMF8$^{-/-}$ pMø at each time point, *****p<0.05; ******p<0.01; *******p<0.001; **#** indicates differences between SLAMF8$^{+/+}$ pMø vs SLAMF8$^{+/+}$ pMø treated with inhibitors. #p<0.05; ##p<0.1; ###p<0.001; **+** indicates differences between SLAMF8$^{-/-}$ pMø vs SLAMF8$^{-/-}$ pMø treated with inhibitors, **+** p<0.05; **++** p< 0.01; **+++** p<0.001. Values indicate mean ± S.E.M. of three independent experiments. C., Control; RDU, relative densitometry units; S, Serine; T, threonine.

Figure S3.

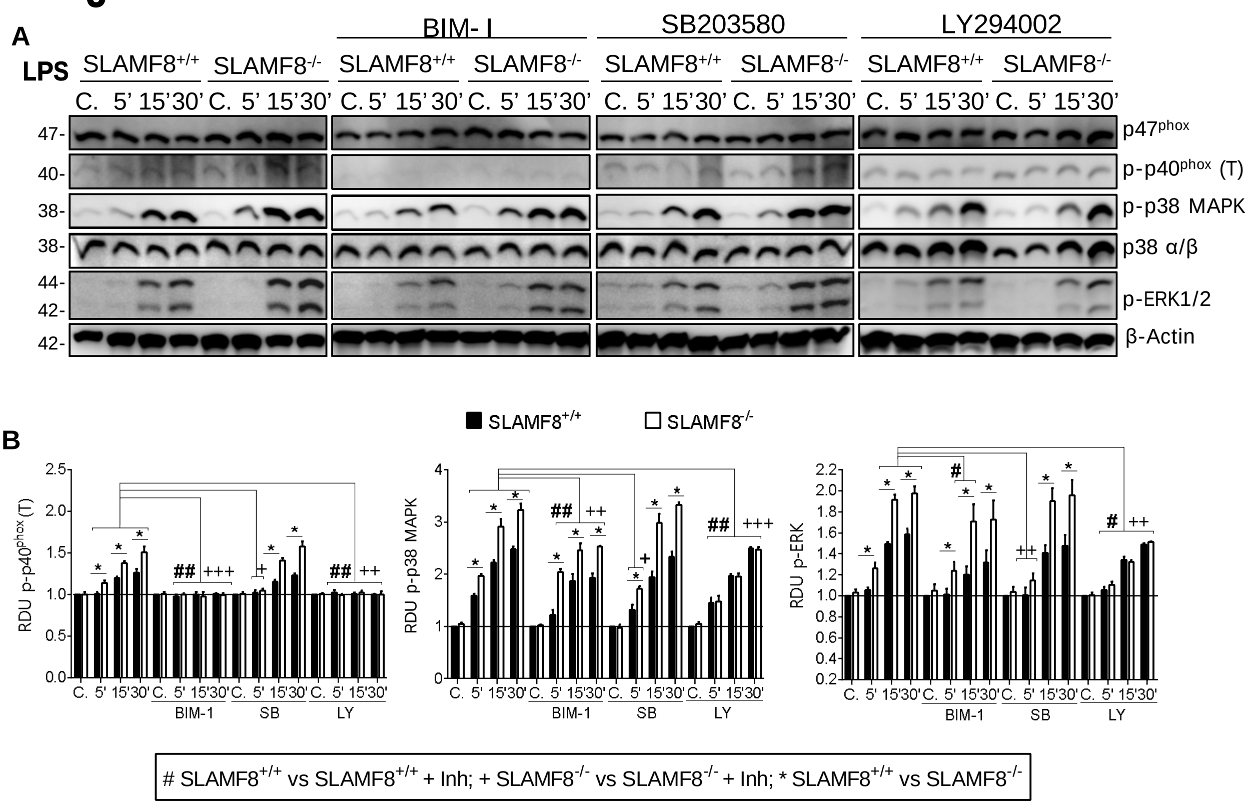

Figure S3. Study of NOX2 activation through phosphorylation analysis of p47^{phox}, p40^{phox}, p38/ERK MAPK in SLAMF8$^{-/-}$ macrophages pretreated with inhibitors and stimulated with LPS. pMø were pretreated with or without 5 µM bisindolylmaleimide I (BIM-1), 10 µM SB203580 (SB), or 10 µM LY294002 (Ly) for 1 h, and stimulated with 100 ng/ml of PMA (A) or 10 µg/ml pure LPS at different time points. **(A)** Representative western blots of the indicated proteins in pMø pretreated with or without the indicated inhibitors and stimulated LPS. **(B)** Relative expression levels of phosphorylated proteins as indicated in A . Levels were normalized to appropriate loading controls and are graphically represented. *****Indicates differences between SLAMF8 $^{+/+}$ pMø vs SLAMF8$^{-/-}$ pMø at each time point, *****p<0.05; *******p*<0.01; ********p*<0.001; **#** indicates differences between SLAMF8$^{+/+}$ pMø vs SLAMF8$^{+/+}$ pMø treated with inhibitors. #*p*<0.05; ##*p*<0.01; ###*p*<0.001; **+** indicates differences between SLAMF8$^{-/-}$ pMø vs SLAMF8$^{-/-}$ pMø treated with inhibitors, **+** *p*<0.05; **++** *p*< 0.01; **+++** *p*<0.001. Values indicate mean ± S.E.M. of three independent experiments. C., Control; RDU, relative densitometry units; S, Serine; T, threonine.

Figure S4.

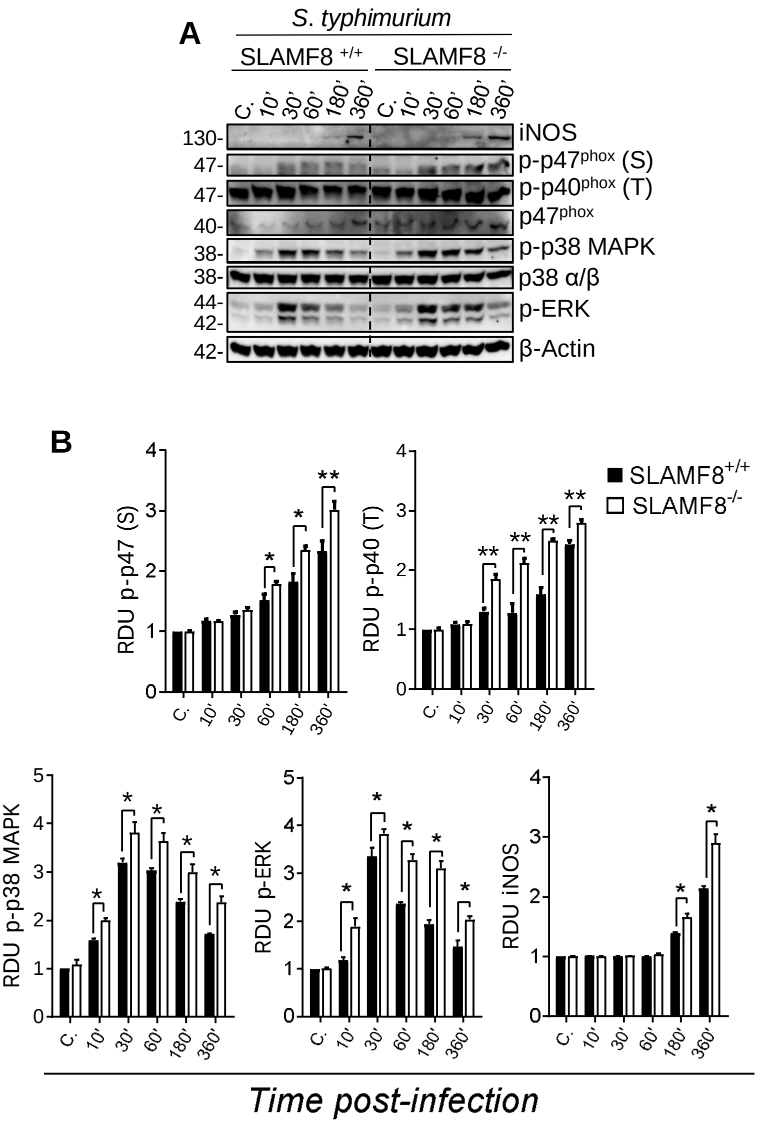

Figure S4. Analyses of activation of NOX2 subunits, ERK1/2 and p38 MAPK in SLAMF8$^{+/+}$ and SLAMF8$^{-/-}$ pMø upon infection *in vitro* with *S. typhimurium.* pMø were infected with *S. typhimurium* (MOI 10) and analyzed at at different time post-infection. **(A)** Representative western blots of indicated protein lysates. Results of one out of three independent experiments is shown. **(B)** Relative expression of phosphorylated proteins indicated in A, levels were normalized to appropriate loading controls and are graphically represented. Values indicate mean ± SEM. *****Indicates differences between SLAMF8 $^{+/+}$ vs SLAMF8$^{-/-}$ cells at each time point, ******p*<0.05; *******p*<0.01, ********p*<0.001. RDU, relative densitometry units; S, Serine; T, Threonine.

Figure S5

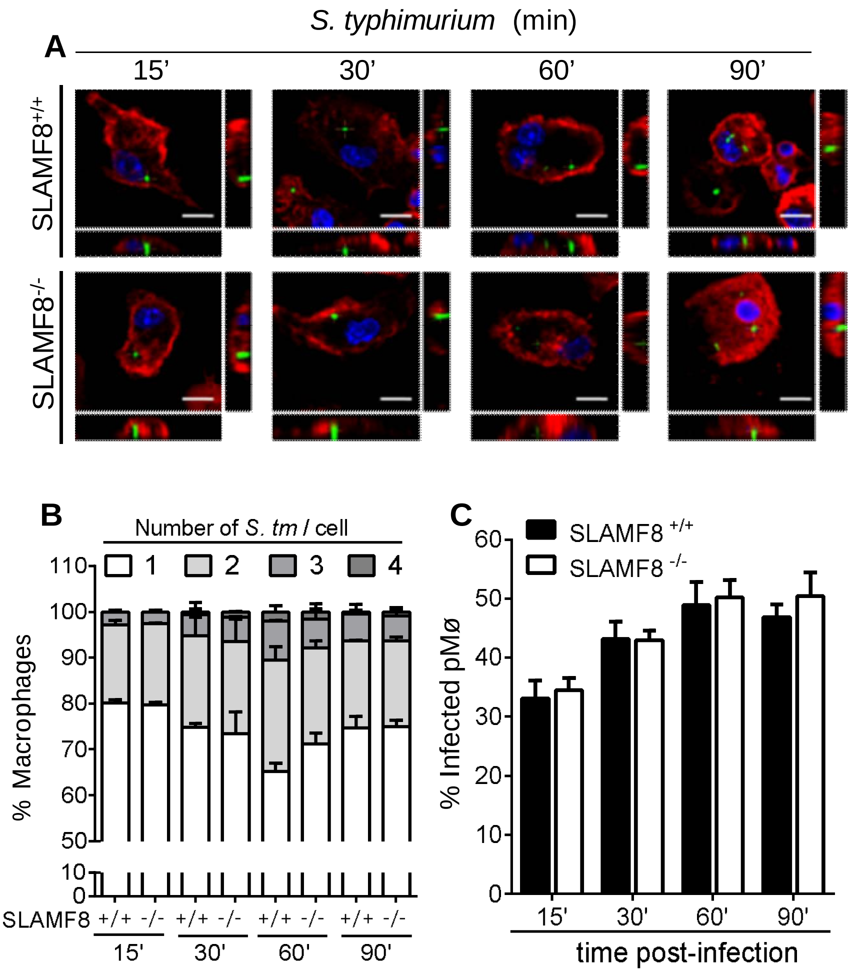

Figure S5. Analysis of phagocytosis in pMø infected with S. typhimurium-GFP by confocal images. pMø were infected with *S. typhimurium-GFP and stained with phalloidin-TRITC (indicates plasma membrane surrounding) to visualize phagocytic bacteria*. Cell nuclei were stained with DAPI. Confocal images (z-projection) and orthogonal views were used to analyze the bacterial uptake, Scale bar: 10 µm (60X). **(D)** Percentage of pMø infected and the number of *S. typhimurium* per cell is indicated. **(E)** Percentage of pMø infected with *S. typhimurium.* Mean ± SEM of three independent experiments is shown (n=100 stochastic cells analyzed per coverslip and experiment). %, percentage; RDU, relative densitometry units; S, Serine; T, Threonine.

Figure S6.

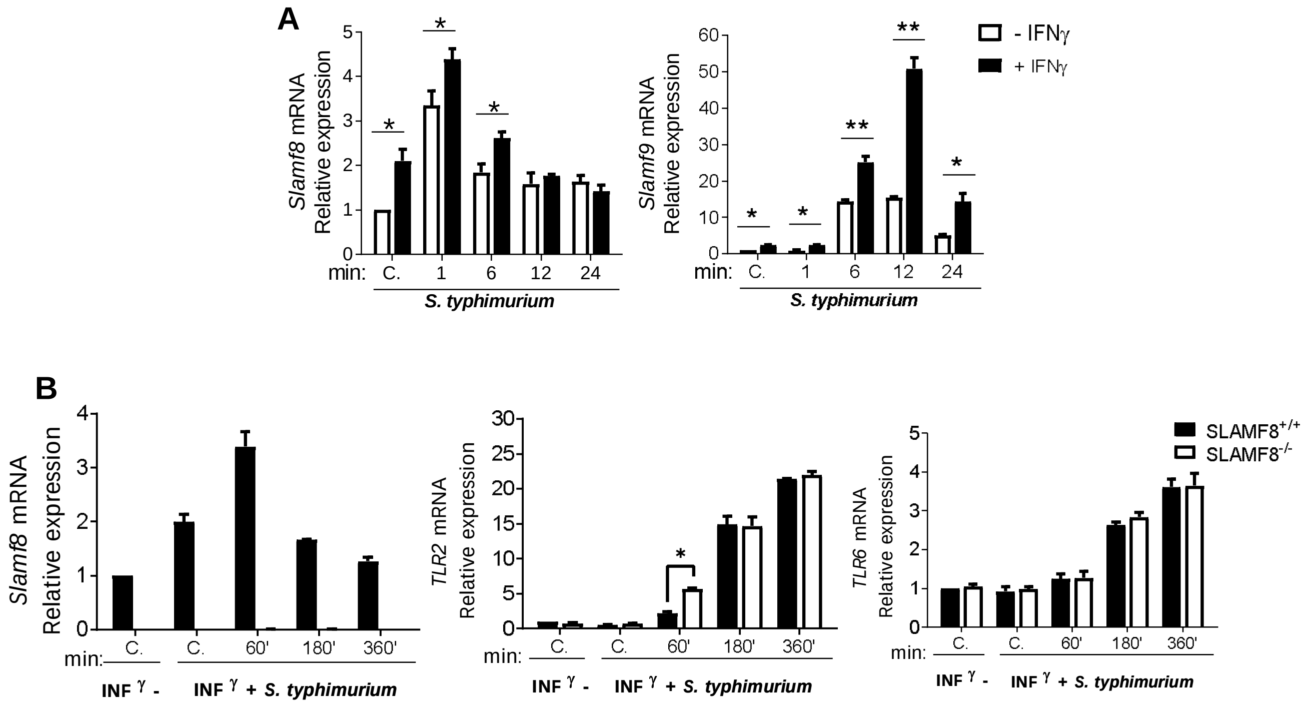

Figure S6. SLAMF8$^{-/-}$ pMø do not show differences in SLAMF9 or TLRs expression. Expression levels of the indicated mRNAs as determined by quantitative RT-PCR in pMø treated with or without IFNγ (100 U/ml) for 16 h, and then infected with *wt S. typhimurium* (MOI 10) at different chase times. **(A)** Expression of *Slamf8* and *Slamf9* in *wt* pMø. **(B)** Expression of the indicated genes in *wt* and SLAMF8$^{-/-}$ pMø. Gene expression was normalized to basal expression levels of untreated cells (C.). Data were analyzed by the $2^{-\Delta\Delta ct}$ method and *HPRT1* was used as the reference gene. Results of two independent experiments are shown. *$*p<0.05$; $**p<0.01$; $***p<0.001$.*

Figure S7.

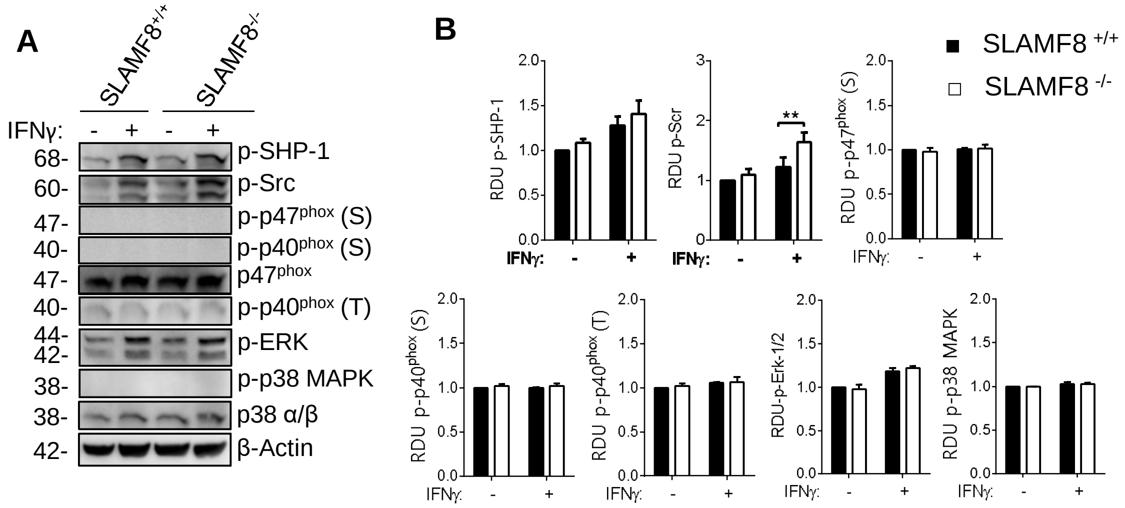

Figure S7. Study of Proteins activation in pMϕ stimulated with IFNγ. Increased phosphorylation of Src. (**A)** Representative western blots (left) of the indicated proteins in pMø treated with or without IFNγ (100 U/ml) for 16 h. (**B**) Graphs represent the relative expression of the indicated proteins (right). Levels were normalized to appropriate loading controls and are graphically represented. Values indicate mean ± SEM of three independent experiments *p<0.05, **p<0.01. RDU, Relative densitometry units; S, Serine; T, Threonine.

Figure S8.

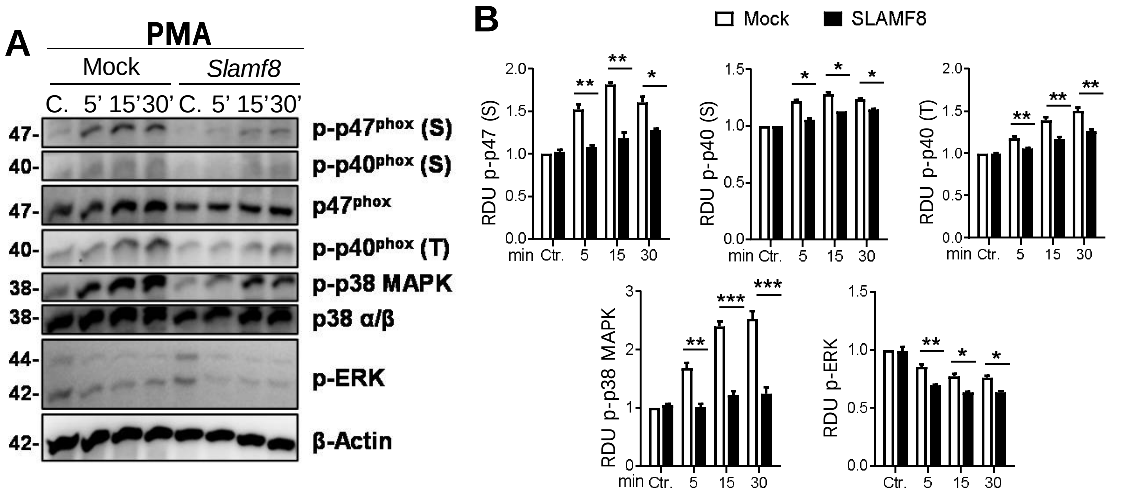

Figure S8. ***Study of NOX2 activation through phosphorylation analysis*** **of p47**[phox]**, p40**[phox] **and p38/ERK MAPK in** ***Slamf8*** **or mock-transfected RAW264.7 Mϕ. Stimulations with PMA.** Stable clones isolated from RAW264.7 Mϕ transfected with cDNA encoding Myc-tagged *Slamf8* (■) or mock transfected (□) were activated with 100 ng/ml PMA at different times. **(A)** Representative western blots of indicated proteins are shown. Results of one out of three independent experiments is shown. **(B)** Relative expression levels of the indicated proteins in A. Relative phosphorylation levels were normalized to appropriate loading control and are graphically represented. Values indicate mean ± SEM of three independent experiments. **p<0.05; **p<0.01; ***p<0.001.* Ctr., control; RDU, relative densitometry units; Ser/S, Serine; Thr/T, Threonine.

Figure S9.

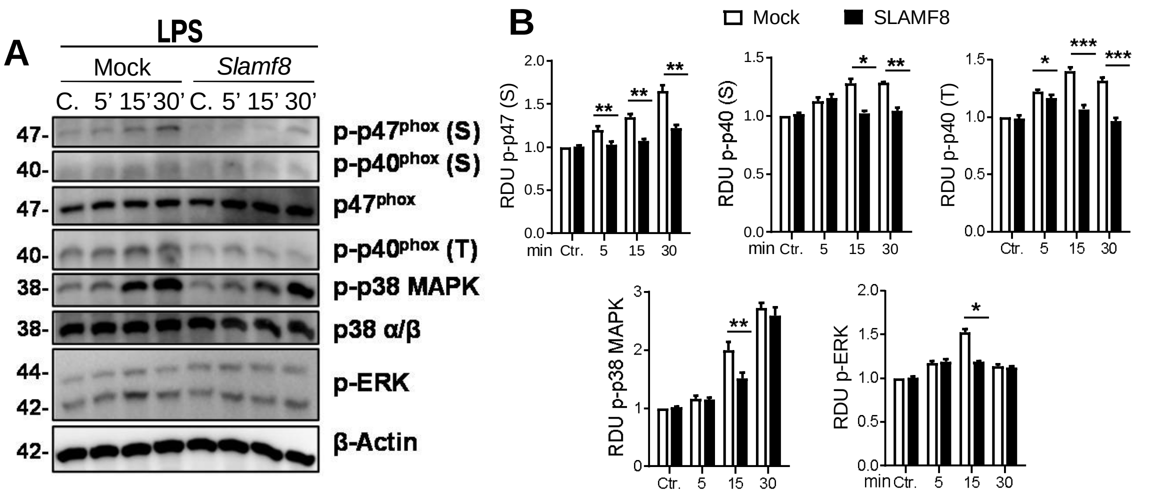

Figure S9. Study of NOX2 activation through phosphorylation analysis of p47^{phox}, p40^{phox} and p38/ERK MAPK in *Slamf8* or mock-transfected RAW264.7 Mϕ. Stimulations with LPS. Stable clones isolated from RAW264.7 Mϕ transfected with cDNA encoding Myc-tagged Slamf8 (■) or mock transfected cells (□) were activated with 10 µg/ml LPS at different times. **(A)** Representative western blots of indicated phosphorylated proteins are shown. Results of one out of three independent experiments are shown. **(B)** Relative expression of the indicated proteins as indicated in A. Phosphorylation levels were normalized to appropriate loading control and are graphically represented. Values indicate mean ± SEM of three independent experiments. **p<0.05; **p<0.01; ***p<0.001.* Ctr., control; RDU, relative densitometry units; Ser/S: Serine; Thr/T, Threonine.
